# ZC3H4, a novel regulator of mitochondrial complex I, impacts prostate stromal cell senescence, attachment, adhesion and anoikis resistance

**DOI:** 10.1038/s41419-025-08027-8

**Published:** 2025-10-21

**Authors:** Teresa T. Liu, Mia J. Carrarini, Livianna K. Myklebust, Kegan O. Skalitzky, Nathalie El-Khoury, Ian J. Sipula, Alexander Chang, Vishal Soman, Ailing Liu, Nnamdi Ihejirika, Uma R. Chandran, Michael J. Jurczak, William A. Ricke, Donald B. DeFranco, Laura E. Pascal

**Affiliations:** 1 Department of Urology, University of Wisconsin, Madison, WI USA; 2 Department of Pharmacology and Chemical Biology, University of Pittsburgh School of Medicine, Pittsburgh, PA USA; 3 Department of Biological Sciences, Carnegie Mellon University, Pittsburgh, PA USA; 4 Molecular Genetics and Developmental Biology Graduate Program, University of Pittsburgh School of Medicine, Pittsburgh, PA USA; 5 Department of Medicine, Division of Endocrinology and Metabolism, University of Pittsburgh School of Medicine, Pittsburgh, PA USA; 6 Department of Biomedical Informatics, University of Pittsburgh, Pittsburgh, PA USA; 7 Department of Immunology, University of Pittsburgh School of Medicine, Pittsburgh, PA USA; 8 Pittsburgh Institute for Neurodegenerative Diseases, University of Pittsburgh School of Medicine, Pittsburgh, PA USA; 9 UPMC Hillman Cancer Center, University of Pittsburgh School of Medicine, Pittsburgh, PA USA

**Keywords:** Cell adhesion, Senescence, Prostatic diseases

## Abstract

Declining mitochondrial function is an established feature of aging and contributes to most aging-related diseases through its impact on various pathologies such as chronic inflammation, fibrosis and cellular senescence. Our recent work suggests that benign prostatic hyperplasia, which is an aging-related disease frequently associated with inflammation, fibrosis and senescence, is characterized by a decline in mitochondrial function. Here, we utilize glycolytic restriction and pharmacologic inhibition of the mitochondrial electron transfer chain complex I to promote mitochondrial dysfunction and identify the cellular processes impacted by declining mitochondrial function in benign prostate stromal cells. Using this model, we show that mitochondrial dysfunction induced alterations in cell-cell and cell-matrix adhesion, elevated fibronectin expression, resistance to anoikis and stress-induced premature senescence (SIPS). We also showed that ablation of ZC3H4, a transcription termination factor implicated in anoikis-resistance and reduced in BPH relative to normal prostates, phenocopied various phenotypes in the human BHPrS1 prostate stromal cell line that resulted from inhibition of complex I. Furthermore, ZC3H4 ablation resulted in the elevation of mitochondrial superoxide (mtROS) and mitochondrial membrane potential, altered mitochondrial morphology and NAD^+^/NADH ratio, and reduced CI function in BHPrS1 cells. Thus, ZC3H4 loss promotes mitochondrial dysfunction to drive pathophysiologic changes in the stromal compartment that are features of the aging prostate.

## Introduction

Benign prostatic hyperplasia (BPH) is a prevalent aging-related condition characterized by benign expansion of both the stromal and epithelial compartments of the prostate and often associated with burdensome lower urinary tract symptoms (LUTS). Aging is the major risk factor for LUTS/BPH, but the pathophysiologic changes attributed to normal and abnormal aging that operate to trigger LUTS/BPH remain largely enigmatic. Impingement of the prostatic urethra by prostatic enlargement contributes to LUTS, but not all patients with LUTS/BPH have enlarged prostates and not all patients respond to the most common pharmacotherapies, which target smooth muscle tone (i.e., alpha-blockers) or prostate growth (i.e., 5-alpha reductase inhibitors). Prostate inflammation and fibrosis are also risk factors associated with LUTS/BPH development and pharmacotherapy resistance, but effective drug therapy targeting these pathologies have not been developed due in large part to our limited understanding of the mechanisms linking inflammation and fibrosis (as well as other pathophysiologic processes) to LUTS/BPH progression.

We recently reported declining mitochondrial function in BPH tissue, particularly in the stromal compartment, which could impact prostatic inflammation and fibrosis, as well as bladder voiding [1]. NDUFS3, a component of complex I (CI) of the electron transport chain (ETC) essential for its efficient function [2], was decreased in the prostates of aging mice and in the prostates of BPH patients [1, 3]. Increasing the levels of prostate NDUFS3 by oleic acid treatment in mice led to an alleviation of lower urinary tract dysfunction [1]. Thus, detailed mechanistic understanding of the factors contributing to declining mitochondrial function in the aging prostate and its consequences could provide additional novel therapeutic targets that could limit LUTS/BPH disease progression and enhance the efficacy of current pharmacotherapies that are focused on alleviation of symptoms.

Due to their specialized secretory function, epithelial cells in the prostate utilize aerobic glycolysis to generate ATP resulting from the Zn^2+^ dependent shunting of citrate to the secretory pathway [4–6]. This metabolic adaptation does not operate in cells within the prostate stroma, which predominantly use oxidative phosphorylation (OXPHOS) to generate ATP [6]. Therefore, declining mitochondrial function in the aging prostate would be expected to have a more pronounced effect on the metabolic stability of stromal cells. A challenge to studying OXPHOS function in vitro is that typical cell culture conditions promote a highly glycolytic metabolic state, where defects in OXPHOS are well tolerated and masked by glycolytic ATP production [7]. However, cultured cells subjected to glucose-deprivation predominantly rely on OXPHOS for ATP production and maintaining metabolic homeostasis and therefore are highly sensitive to disruptions in OXPHOS [8]. Specifically, inhibition of OXPHOS in cells with limited glycolytic activity results in decreased levels of NADPH and glutathione (GSH) and increased oxidative stress that can lead to cell death and the generation of a pro-inflammatory state [9]. The disruption of OXPHOS has been implicated as a mechanism contributing to idiopathic pulmonary fibrosis and cardiovascular fibrosis [10].

In the current study, we utilized an established paradigm for glycolytic restriction (i.e., replacing glucose with galactose as the sole 6 carbon sugar energy source) in the human BHPrS1 benign prostate stromal cell line to reveal the impact of OXPHOS disruption on prostate stromal cell function. Glycolytic restricted BHPrS1 cells are more reflective of the metabolic state of prostate stromal cells in vivo and their reliance on OXPHOS for maintaining ATP levels and metabolic homeostasis makes them more susceptible to mitochondrial stress elicited by pharmacologic disruptions of the ETC, particularly in CI. Our results provide the first demonstration of the impact of mitochondrial function on maintaining prostate stromal cell homeostasis, particularly their cell-cell and cell-matrix adhesion, resistance to anoikis and restricted senescence. Furthermore, we identified ZC3H4, a zinc finger transcription termination factor that maintains the integrity of genome-wide non-coding intergenic transcription, as essential for maintaining prostate stromal cell homeostasis and CI function, as well as a new biomarker associated with metabolic stress and with altered expression in select prostatic cell types in BPH patients.

## Materials and methods

### Cell culture

Simon Hayward (Northshore Research Institute) provided the BPH-1 human prostate epithelial cell line [11], and the BHPrS1 human benign prostate stromal cell line, which share characteristics of activated fibroblasts based upon their expression of vimentin and ɑ-smooth muscle actin [12]. Primary human BPH prostate stromal cells TP18S317, which express vimentin, ɑ-smooth muscle actin and calponin [13], were derived from patients with BPH and kindly provided by Zhou Wang (University of Pittsburgh, IRB#20090026). Experiments were conducted using BHPrS1 and BPH-1 cells between passage number 10 to 50, and TP18S317 cells between passage number 5 to 15. The genetic identity of BPH-1 and BHPrS1 cell lines were authenticated in 2016 using DNA fingerprinting by examining microsatellite loci in a multiplex PCR (AmpFlSTR Identifiler PCR Amplification Kit, Applied Biosystems, Foster City, CA) by the University of Pittsburgh Cell Culture and Cytogenetics Facility. Cell lines were confirmed as mycoplasma free by DNA staining with DAPI or Hoechst.

### Glycolytic restriction and mitochondrial complex I inhibition

Cells were grown in RPMI-1640 containing 11.11 mL-glucose (Cat# 11875119, Gibco, ThermoFisher, Waltham, MA, USA) supplemented with 10% FBS and 1% P/S, or RPMI-1640 Cat# 11879020, Gibco) lacking glucose but supplemented with 10 mM galactose (CAS#59-23-4, Cat# G0750, Sigma-Aldrich, St. Louis, MO, USA), 10% FBS, and 1% P/S at 37 °C and 5% CO_2_ in a humidified incubator. For experiments modeling OXPHOS disruption, all cells were plated in glucose-containing media overnight and then switched to fresh glucose-or galactose-containing media. Media was again refreshed on days three and four. On day four, cells were treated with 10–1000 nM rotenone (CAS#83-79-4, Cat#13995, Cayman Chemical, Ann Arbor, MI, USA) or DMSO vehicle control in glucose- or galactose-containing media for a further 24 to 72 h. Rotenone and DMSO were freshly prepared immediately before each use.

### Cell proliferation, ATP and cell doubling time assays

For cell number and proliferation assays, 1.0 × 10^5^ cells were seeded in 6-well plates with at least three wells for each condition. Cells were incubated in glucose- or galactose-containing media with or without rotenone (DMSO vehicle control or 50 nM rotenone) for the indicated times and were trypsinized and counted using a trypan-blue exclusion assay to quantify viable cells (Cellometer Auto2000, Nexcelom Biosciences, Lawrence, MA, USA). Intracellular ATP levels were assayed using a CellTiter-Glo Luminescent Cell Viability Assay (Cat# G7570, Promega, Madison, WI, USA). Cells were seeded in 96-well plates at 5.0 × 10^3^ cells/well with at least six wells plated for each condition. Luminescence was measured using a SpectraMax iD5 plate reader (Molecular Devices, San Jose, CA, USA). Cell doubling time was determined by seeding 2.5 × 10^5^ cells in T75 flask with at least three plates for each condition and each timepoint. Cells were trypsinized and counted after 48 or 72 h. Doubling time was calculated using the Cell Doubling Time Calculator (https://www.omnicalculator.com/biology/cell-doubling-time). Each experiment was repeated a minimum of three times.

### Flow cytometric apoptosis and cell cycle analysis

Sub-confluent cells (seeded at a density of 2.5 × 10^5^ cells/T75 flask) were treated with and without glycolytic restriction for three days, followed by rescue from glycolytic restriction or with and without rotenone or DMSO vehicle control for an additional 24 h and a minimum of 1.0 × 10^6^ cells were collected for each condition. Collected cells were stained with FxCycleTM Violet Stain (Cat# F10347, Molecular Probes, Eugene, OR, USA) to measure DNA content for cell cycle analysis or Dead Cell Apoptosis Kit with Annexin V Alexa FluorTM 488 and Propidium Iodide (Cat# V13241, Invitrogen, Life Technologies Corporation, Eugene, OR, USA) and fluorescence data acquisition was performed using the Fortessa 7S (BD Biosciences, Franklin Lakes, NJ, USA) cell sorter in the University of Pittsburgh School of Medicine Unified Flow Cytometry Core. Cell cycle experiments were repeated two times and apoptosis analysis was completed once. Apoptosis analysis of siNC and siZC3H4 cells under glycolytic conditions was performed three times.

### Sudan Black B staining

Sudan Black B (CAS#4197-25-25, Cat#199664, Sigma-Aldrich) histochemical staining for lipofuscin to detect senescent cells was performed as described [14]. Cells were seeded in 12-well plates at 1.5 × 10^5^ cells per well with three replicates per well. Briefly, following treatment, cells were fixed in 4% PFA for 5 min at room temperature. Cells were then washed three times with PBS before fixing in 70% ethanol for 2 min. Cells were stained with freshly prepared and filtered Sudan Black B (15 mM in 70% ethanol), rinsed in 50% ethanol, and washed in distilled water before imaging. Quantitation of staining was performed by counting the number of stained cells per 20× magnification image for a minimum of three non-overlapping images per well and results were expressed as the percentage of stained cells per total number of cells. Experiments were repeated a minimum of three times.

### Quantitative real-time polymerase chain reaction (qRT-PCR)

RNA was isolated from BHPrS1 cells after treatment using TriZol (Invitrogen) and Direct-zol RNA MiniPrep Plus kit (Cat# R2072, Zymo Research, Irvine, CA, USA). cDNA synthesis was performed using the iScript cDNA Synthesis Kit (Bio-Rad). qRT-PCR was performed using the CFX96 Touch Real Time Detection System with products generated using iTaq Universal SYBR Green Supermix (Bio-Rad). Gene-specific primers were listed in Supplementary Table S1. Each experiment was repeated a minimum of three times with three biological and two experimental replicates.

### Immunocytochemistry/Immunofluorescence (ICC/IF)

IF staining was performed on fixed cells using antibodies listed in Supplementary Table S2. DNA was stained with Hoechst 33258 (1:1000, Cat # A33258, Sigma-Aldrich). Briefly, cells were fixed in 4% PFA for 15–30 min, washed in PBS and blocked in 10% normal goat serum (NGS), 0.1% Triton X-100 in PBS for 30 min. Cells were then incubated in primary antibodies in 10% NGS overnight at 4 °C. Cells were washed and incubated with the appropriate secondary antibodies (in 10% NGS in PBS) for one h and Hoechst for 1 min. Stained sections were imaged with an Olympus BX51 (Olympus, Center Valley, PA, USA) equipped with an F-View II camera (Olympus) and DP-BSW software (Olympus), or an Olympus IX71 inverted microscope (Olympus) equipped with a DP30BW camera (Olympus) and DP-BSW software (Olympus). Microscope settings were kept constant throughout imaging and staining intensity was determined using PhotoShop [15]. Cell area was determined using ImageJ [16]. Each staining experiment was repeated a minimum of three times with three biological replicates per experiment.

### Quantitation of detached and adherent cells

Quantitation of cells adhering to the culture plate and those detached and suspended in the media were determined as the % of total viable cells (adherent + detached) as previously described [17]. Briefly, media was aspirated from cell culture plates and centrifuged to generate a cell pellet to collect detached cells. The remaining adherent cells were rinsed twice in PBS, then trypsinized and centrifuged to generate a cell pellet. Pellets were resuspended and detached and adherent cells were separately counted using a trypan blue exclusion assay and quantified using a Cellometer (Nexcelom Bioscience). Cells were imaged with an Olympus IX71 inverted microscope (Olympus). The mean number of cells in aggregated clusters was determined by counting the number of cells in each cluster for each treatment condition, a minimum of 10 clusters were counted per condition. At least three wells were plated for each condition and each experiment was repeated a minimum of three times.

### Colony formation assay of detached cells

Detached cells were pelleted using centrifugation and dispersed by gentle repeated pipetting. Cells were seeded at 1 × 10^3^ cells/well into 12-well plates supplemented with glucose media (under glycolytic conditions) and colony formation was assessed six days after re-seeding [17]. Cell colonies were then rinsed twice in PBS, fixed in 4% PFA overnight, then stained with 0.1% methylene blue for at least 20 min. Stained cells were then washed with DI H_2_O and allowed to dry at room temperature. Images were captured using a LI-COR Odyssey DLx fluorescent imager (LI-COR, Lincoln, NE, USA). Colonies were counted using Adobe PhotoShop (PhotoShop, v. 25.6.0, Adobe, San Jose, CA, USA). At least three wells were plated for each condition and experiments were repeated a minimum of three times.

### Cell-type specific expression of ZC3H4 mRNA in normal and BPH prostate

Prostate normal and BPH cell-type specific mRNA expression of ZC3H4 was obtained from publicly available single cell RNA-Seq datasets: GEO GSE145843 (human normal) and GSE145838 (human BPH) [18]. Data were downloaded from the CellxGene database in the form of a Seurat object (loaded using Seurat v5.1). The object contained 9 samples, which included a total of 28,847 cells and 22,644 genes. The Seurat object was subset to include 6 samples of interest: 3 normal and 3 BPH samples, reducing the dataset to 19,211 cells. The subset Seurat object was then used to generate a violin plot, illustrating the distribution of gene expression across cell populations, stratified by disease status.

### ZC3H4 ICC/IF staining of prostate TMA

Immunofluorescent staining of a prostate tissue microarray (TMA) including BPH and age-matched normal prostate [19] was performed according to OPAL manufacturer protocols (Akoya Biosciences, Marlborough, MA). Sections were deparaffinized and rehydrated, and antigen retrieval was performed using Tris-EDTA pH 9.0. The TMA was stained with anti-ZC3H4 (20041-1-AP, Proteintech). Images were acquired using the Mantra Scope System (Akoya Biosciences) and quantified using InForm software (Akoya Biosciences). An unstained prostate section was processed simultaneously with multiplex samples and imaged to quantify autofluorescence. Cell and tissue segmentation was performed to examine protein localization. Optical density was quantified for each antibody and each image was segmented into cells based on DAPI nuclear staining. Quantitation of staining intensity was acquired for epithelial (Normal (*n* = 30) and BPH (*n* = 6)) and stromal (Normal (*n* = 28) and BPH (*n* = 6)) ZC3H4 staining in human prostate tissues. Tissue sections that were washed away during rinsing were not included in the analysis.

### RNA interference targeting ZC3H4

BHPrS1 cells were transiently transfected with dicer-substrate ZC3H4-targeting and non-coding control siRNAs (Cat# hs.Ri.ZC3H4.13, TriFECTa RNAi kit, IDT Technologies, Coralville, IA, USA) using DharmaFECT 1 transfection reagent (Cat# T-2001-03, Horizon, Lafayette, CO, USA) in serum-free and antibiotics-free media. The following dsiRNAs from IDT were utilized for knockdown experiments either individually, or as a pool: siZC3H4 #1, 5’GACUACGAGAAUGAGCAGUAUGGGG-3’ and 3’-UCCUGAUGCUCUUACUCGUCAUACCCC; and siZC3H4 #3, 5’-AGCAUCACAGUGAUUCGGAUGAGGA-3’ and 3’-CUUCGUAGUGUCACUAAGCCUACUCCU-5’. Knockdown of ZC3H4 was confirmed by qRT-PCR. Primer sequences are listed in Supplementary Table S1.

### Mitochondrial membrane potential

Mitochondrial membrane potential (MMP) was measured using the JC1- Mitochondrial Membrane Potential Assay Kit ab113850 (Cat# ab113850, Abcam). Treated cells were seeded at 1.5 × 10^4^ cells/well in 96-well black wall/clear bottom plates for 24 h. Upon completion of the treatment, the supernatant was aspirated, and cells were incubated with 100 μl of 1 μM JC-1 in medium in each well for 15 min at 37 °C. Cells were then washed twice with PBS and dilution buffer, and the fluorescence intensities of JC-1 monomers and aggregates were measured using a SpectraMax iD5 plate reader (Molecular Devices, San Jose, CA). JC-1 monomers were detected at wavelength of 485 nm for excitation and 535 nm for emission, whereas JC-1 aggregates were detected at wavelength of 530 nm for excitation and 590 nm for emission. MMP was measured as the ratio of JC-1 aggregate to monomer, and the results in treated cells were expressed as percentage of the results in DMSO controls. FCCP 100 µM treatment for 4 h was utilized as an uncoupling agent. Cells in wells that were washed away during rinsing were not included in the results. The experiment was repeated three times with 16 replicates per condition.

### Western blotting for ZC3H4 using concentrated cell lysates

Ammonium sulfate protein precipitation was used to concentrate the BHPrS1 lysate protein for ZC3H4 Western blot analysis following knock down. T-75 flasks containing 70% confluent BHPrS1 cells following siNC or siZC3H4 knockdown were lysed in detergent-free buffer (50 mM Tris-HCl, 150 mM NaCl, pH 8.0) containing HALT protease and phosphatase inhibitor cocktail (Cat# 78442, ThermoFisher Scientific, Waltham, MA), yielding approximately 1 ml lysate per flask. Saturated ammonium sulfate (4.1 M) was added dropwise to 1 ml of lysate and subsequently incubated at 4 °C with gentle agitation. Protein was pelleted by centrifugation at 12,000 × *g* for 30 min at 4 °C and resuspended in 30 μl RIPA buffer containing HALT. Supernatants were collected for iterative fractionation. Samples were sequentially precipitated at 20% followed by 30% ammonium sulfate saturation. Protein concentrations were determined using BCA assay (# 23225, ThermoFisher Scientific, Waltham, MA). The 30% fractionated lysates from siNC and siZC3H4 conditions were used for Western blot analysis.

All other samples were prepared by lysing 70% confluent BHPrS1 cells following siNC or siZC3H4 knockdown in RIPA buffer (50 mM Tris HCl, 1% Triton X-100m 0.5% Sodium Deoxycholate, 0.1% SDS, 150 mM NaCl) containing the HALT protease and phosphatase inhibitor cocktail followed by 1 × 10 sec sonication pulse. As above, protein concentrations were determined using BCA assay (Cat#23225, ThermoFisher Scientific, Waltham, MA).

Sample lysates (15 μg/well) were loaded into Mini PROTEAN TGX polyacrylamide gels (4561093, Biorad) alongside Precision Plus Protein Dual Color Standards (1610374, Bio-Rad Laboratories, Hercules, CA). Proteins were separated by SDS gel electrophoresis and transferred to PVDF membranes (IPFL00010, Millipore). After transfer, PVDF membranes were stained with the Li-Cor Revert™ 700 Total Protein Stain Kit (926-11010, LiCorBio, Lincoln, NE, USA) for protein normalization and imaged using a Li-Cor Odyssey Dlx. The membranes were then blocked in 5% milk and probed with the primary antibodies (Supplementary Table S2) at 4 °C overnight. Membranes were secondarily probed with IRDye 680 goat anti rabbit (926-68071, LiCorBio) and IRDye-800 goat anti mouse (926-32210, LiCorBio) and imaged with a Li-Cor Odyssey Dlx. Protein level quantification relative to total protein was carried out using LiCor Image Studio™ software. All western blots were repeated three times on three biological replicates.

### Mitochondrial superoxide

BHPrS1 cells treated with siNC or siZC3H4 knockdown were trypsinized and seeded at 2 × 10^4^ cells/well overnight in a black clear-bottom 96-well plate (Cat#3603, Corning) in galactose supplemented media. To optimize response to rotenone, BHPrS1 cells seeded at 2 × 10^4^ cells/well overnight were treated with fresh media containing vehicle control, 10 nM rotenone or 100 nM rotenone for 3 h, then stained with 500 nM MitoSOX (M36008, Invitrogen) for 30 min. For knockdown cells, media was replaced with fresh media containing vehicle control or 10 nM rotenone for 3 h before MitoSOX staining. Cells were then washed gently three times with warm HBSS and mtROS was detected using a plate reader at a wavelength of 510 nm for excitation and 580 nm for emission. Cells in wells that were washed away during rinsing were not included in the analysis. The experiment was repeated three times with 6 replicates per condition.

### Mitochondrial network morphology

BHPrS1 cells treated with siNC or siZC3H4 knockdown were trypsinized and seeded at 2 × 10^4^ cells/chamber on chamber slides overnight. Mitochondria were either stained with MitoTracker (M7512, Invitrogen), or fixed in 4% PFA for 20 min then stained with TOM20. Images were acquired using an Olympus BX51 fluorescence microscope, equipped with F-View Soft Imaging System. Mitochondrial morphology was quantitatively assessed using ImageJ and the MiNA (Mitochondrial Network Analysis) utility [20]. This experiment was repeated twice and data reflects assessment of 45 individual siNC cells and 67 siZC3H4 cells.

### NAD^+^/NADH assay

Determination of NAD^+^ and NADH and NAD^+^/NADH ratio was performed using the NAD^+^/NADH Assay kit (MAK460, Sigma-Aldrich). BHPrS1 cells were grown in glucose, galactose, or galactose plus siZC3H4 knockdown for 48 h. Treated cells were washed with cold PBS and pelleted at 1 × 10^6^ cells per sample then homogenized in either NAD^+^ or NADH extraction buffer and heated for 5 min at 60 °C. Cells were vortexed then centrifuged for 5 min and supernatant was transferred to a black clear-bottom 96-well plate for analysis. NAD^+^ and NADH were detected using a plate reader at a wavelength of 530 nm for excitation and 585 nm for emission. This experiment was repeated once with three biological replicates per condition.

### Mitochondrial respirometry of siZC3H4 and siNC BHPrS1 cells

Mitochondrial respirometry was performed on BHPrS1 cells treated with siRNA knockdown of ZC3H4 (siZC3H4) or non-coding control (siNC) using an Oroboros O_2_K High-resolution Respirometer (Oroboros, Innsbruck, Tyrol, Austria). BHPrS1 cells were plated at 2 × 10^6^ cells in T75 flasks in glucose-containing media overnight, then transiently transfected with non-coding control siRNA or a pool of siZC3H4 #1 and siZC3H4 #3 siRNAs in serum-free media supplemented with galactose for 48 h. Media was then replaced with galactose-supplemented media overnight. Treated cells were trypsinized and resuspended in ice-cold MiRO5 buffer at 4 × 10^5^ cells/ml. Mitochondrial respiration was measured using a total of 8 × 10^5^ cells per chamber. Cells were permeabilized in chamber with a predetermined dose of digitonin (4 μM) that was the minimal required concentration required to achieve cell membrane permeability without compromising outer mitochondrial membrane integrity, which was assessed by cytochrome c (10 μM) addition. Two respirometry protocols were employed to assess complex I mediated respiration and were carried out using serial titrations of the following substrates and inhibitors: (1) pyruvate (5 mM), malate (2 mM), glutamate (10 mM), ADP (2 mM), FCCP (0.5 μM titrations until max respiration achieved), and rotenone (0.5 μM); (2) palmitoyl-carnitine (25 μM), malate (2 mM), glutamate (10 mM), ADP (2 mM), FCCP (0.5 μM titrations until max respiration achieved), and rotenone (0.5 μM). Experiments were performed in duplicate and repeated three times.

### Statistical analysis

Comparisons between groups were calculated using the Student’s *t*-test, Brown–Forsythe and Welch one-way ANOVA with Welch’s correction, or two-way ANOVA with Bonferroni’s multiple comparison test as appropriate. Mitochondrial respirometry comparisons were calculated using a paired Student’s *t*-test according to the day of analysis. A *p*-value of *p* < 0.05 was considered significant. GraphPad Prism version 10 or higher was used for graphics (GraphPad Software, San Diego, CA, USA). Values are expressed as mean ± S.D.

## Results

### Glycolytic restriction of human prostate stromal cells inhibits growth and increases sensitivity to CI inhibition without promoting cell death

**Fig. 1:**
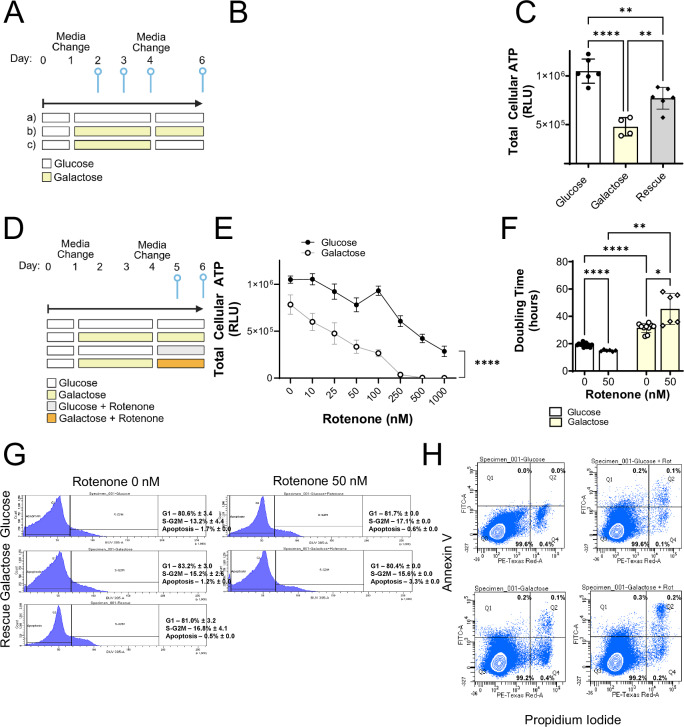
Glycolytic restriction in prostate stromal cells reduces proliferation and ATP and increases sensitivity to rotenone induced mitochondrial ETC C1 inhibition. **A** Treatment paradigm for glycolytic restriction of BHPrS1 prostate stromal cells. Media was refreshed with either glucose or galactose media on days 1, 3 and 4. Created in BioRender. Pascal, L. (2025) https://BioRender.com/8nukelm**B** Cell growth of BHPrS1 cells cultured in (a) glucose, (b) galactose or (c) galactose followed by glucose rescue conditions. Cells were counted via trypan blue exclusion assay on days 2, 3, 4 and 6. **C** Quantitation of ATP concentration in BHPrS1 cells on Day 6 in glucose, galactose, or rescue conditions via CellTiter luminescence assay. **D** Treatment paradigm for glycolytic restriction (galactose) plus CI inhibition via rotenone (0–1000 nM) or DMSO vehicle control of prostate stromal cells. Created in BioRender. Pascal, L. (2025) https://BioRender.com/8nukelm**E** Quantitation of ATP in BHPrS1 cells on Day 6 in glucose, galactose, +/− rotenone culture conditions via CellTiter luminescence assay. **F** Cell doubling time of treated BHPrS1 cells via trypan blue exclusion assay. **G** Representative flow cytometry chart of cell cycle analysis of cells grown in glucose, galactose, rescue and with 0 or 50 nM rotenone (24 h) on Day 5. **H** Flow cytometry chart of apoptotic (Q1), dead apoptotic (Q2), live (Q3) and dead (Q4) BHPrS1 cells on Day 5. Annexin-V/PI-positive apoptotic cells maintained in glucose or galactose and treated with 0 or 50 nM rotenone on Day 5. **H** *p < 0.05; **p < 0.01, ****p < 0.0001.

To model mitochondrial stress under physiological growth conditions, prostate stromal cells were pre-cultured in cell culture medium supplemented with either glucose or galactose, then treated with rotenone, a validated inhibitor of CI [21, 22]. The impact of glycolytic restriction alone on the growth characteristics of BHPrS1 cells was examined over a six-day time-course with either glucose or galactose as the sole 6-carbon energy source on day one with media refreshed on day four (Fig. 1A). Cell proliferation was significantly but reversibly reduced by glycolytic restriction (galactose) over the six-day time-course (Fig. 1B), with cell doubling time restored by 72 h following return to glycolytic conditions (rescue) (Supplementary Fig. S1). Total cellular ATP levels were also significantly but reversibly reduced after 48 h in galactose (Fig. 1C). Decreased ATP levels were also observed in primary human stromal cell cultures derived from a BPH patient [13] (TP18S317) and maintained in galactose compared to glucose; ATP levels were increased after 48 h of glucose substitution for galactose but not restored to the same levels as cells continually exposed to glucose, similar to BHPrS1 cells (Supplementary Fig. S1B). The doubling time of the BPH patient-derived stromal cells was 61.6 ± 19.0 h under glycolytic conditions, which likely reduced the restoration of ATP observed at 48 h with rescue compared to the relative level of ATP restoration in BHPrS1 cells, which had a doubling time of 19 h (see Supplementary Fig. S1A).

BHPrS1 cells were grown in either glucose- or galactose-supplemented media for four days followed by treatment with rotenone (0–1000 nM) to trigger OXPHOS impairment [23] or DMSO vehicle control for 48 h (Fig. 1D). Under glycolytic conditions, BHPrS1 cells were well tolerant of rotenone at lower concentrations ( ≤ 100 nM), while glycolytic restriction in combination with rotenone induced a significant decrease (*p* < 0.0001) in ATP levels in a dose-dependent manner over a range of 10–1000 nM rotenone (Fig. 1E). BHPrS1 cell doubling time was also significantly increased under glycolytic restriction conditions with and without rotenone (50 nM) at 48 h (Fig. 1F). Similarly decreased ATP levels in response to glycolytic restriction and rotenone were observed in primary cultures of stromal cells derived from BPH patient TP18S317 subjected to glycolytic restriction and rotenone treatment (50 nM) (Supplementary Fig. S1C).

Relatively high doses (100–1000 nM) of rotenone are cytotoxic to fibroblasts [24, 25]. To ensure that glycolytic restriction coupled with 50 nM rotenone was not promoting cell death, cell cycle and apoptosis analyses were performed using flow cytometry after 24 h of rotenone treatment. Asynchronous cell cycle phase distribution of sub-confluent prostate stromal cells analyzed by flow cytometry showed that glycolytic restriction with or without rotenone showed no significant difference in the distribution of cells in both S and G2/M phases compared to cells grown in glucose (*p* = 0.53) (Fig. 1G). Apoptosis analysis using Annexin V and propidium iodide staining of BHPrS1 cells revealed no increase in apoptosis in BHPrS1 cells treated with glycolytic restriction and/or rotenone (50 nM) (Fig. 1H). Cumulatively, these data suggest that cell proliferation and ATP generation in BHPrS1 cells under glycolytic restriction were significantly sensitized to sub-cytotoxic levels of rotenone (i.e., 50 nM) without altering cell cycle dynamics or inducing a significant increase in apoptosis compared to CI inhibition under glycolytic conditions. Thus, cell cycle arrest and/or very minor changes in apoptosis are unlikely to account for the dramatic changes in proliferation and cell viability elicited in BHPrS1 cells upon glycolytic restriction in the presence or absence of rotenone.

### Stress-induced premature senescence induced by inhibition of mitochondrial CI

**Fig. 2:**
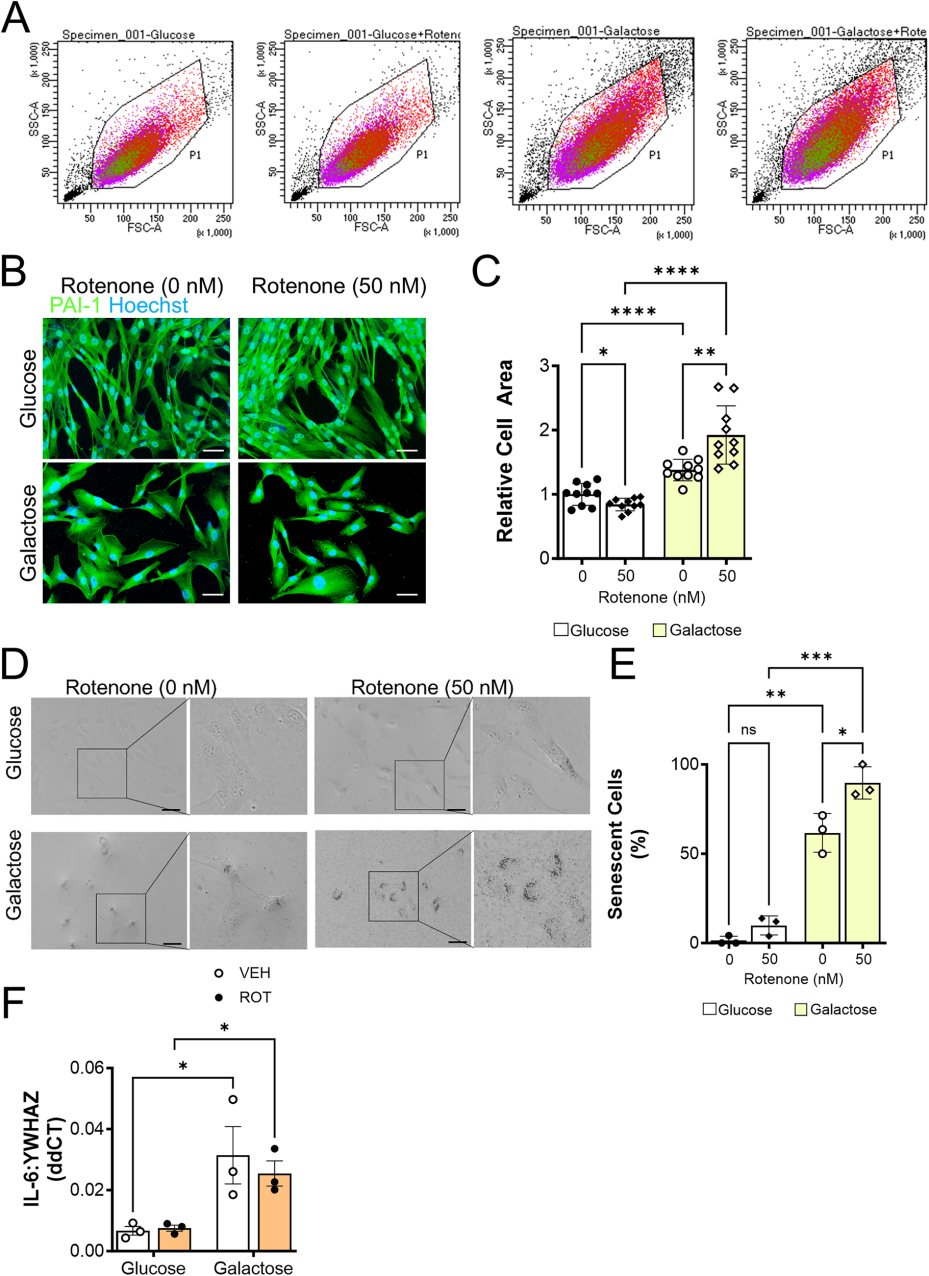
Mitochondrial ETC C1 inhibition coupled with glycolytic restriction induces a senescence phenotype in prostate stromal cells. **A** Flow cytometry analysis of forward (FSC-A) and side (SSC-A) scatter in BHPrS1 cells +/− rotenone (50 nM) or DMSO vehicle control in glucose or galactose culture conditions on Day 5. **B** Impact of glycolytic restriction and rotenone on cellular morphology of BHPrS1 cells on Day 6. Immunofluorescent staining of BHPrS1 cells with PAI-1 (green). Nuclei (blue) stained using Hoechst. Scale bar, 50 µm. **C** Relative cell area as measured by ImageJ analysis. **D** Sudan Black B staining of lipofuscin in BHPrS1 cells following treatment (Day 5). Scale bar, 50 µm. **E** Quantitation of the percentage of Sudan Black B positive senescent cells following treatment. **F** Impact of glycolytic restriction and rotenone on the expression of SASP cytokine IL-6 as determined by qRT-PCR on Day 5. *p < 0.05; **p < 0.01; ***p < 0.001; ****p < 0.0001, ns non-significant.

In addition to affecting proliferation and ATP levels, glycolytic restriction and rotenone impacted the shape and size of BHPrS1 cells. Specifically, forward- and side-scatter flow cytometry analysis of BHPrS1 cells grown under glycolytic restriction conditions revealed an increase in cell size and granularity that was further enhanced by rotenone compared to glycolytic conditions (Fig. 2A). BHPrS1 cells grown in the presence of glucose displayed a regular spindle-shaped morphology characteristic of the phenotype of prostate stromal fibroblasts in typical cell culture conditions (i.e., high glucose). In contrast, BHPrS1 cells grown in the presence of galactose were more flattened and displayed a more sprawling ‘fried egg-like’ shape, characteristic of myofibroblasts (Fig. 2B). In agreement with the increased forward and side-scatter under glycolytic restriction and with rotenone, the total cell area was significantly increased under galactose conditions alone and with rotenone (Fig. 2C). The acute increase in BHPrS1 cell area is reminiscent of cellular morphology changes that accompany the acquisition of a senescent phenotype [26]. Sudan Black B (SSB) staining of lipofuscin, which is a cellular marker of aging and senescence [14, 27], revealed an increase in the percentage of SBB-positive adherent senescent cells under glycolytic restriction that was further increased by rotenone (Fig. 2D, E). Cells undergoing senescence also develop a senescence associated secretory phenotype (SASP), characterized by an increased secretion of proinflammatory cytokines such as IL-6, IL-1β and TNF-ɑ [28]. Elevation of IL-6 has also been associated with BPH progression [29]. IL-6 was increased by glycolytic restriction but not altered by rotenone (Fig. 2F). Although IL-1β demonstrated a similar increased trend, the results were not significant and TNF-ɑ was not altered (Supplementary Fig. S2). Thus, in BHPrS1 cells, rotenone coupled with CI inhibition induced characteristics associated with stress-induced premature cellular senescence (SIPS) and not replicative senescence [30, 31], particularly since they maintain proliferative capacity (even if limited). These results are consistent with the previously reported model of aging dermal fibroblasts that demonstrated rotenone induction of a pre-replicative SIPS phenotype [25].

### BHPrS1 cell attachment to the substratum is reduced by glycolytic restriction coupled with CI inhibition

**Fig. 3:**
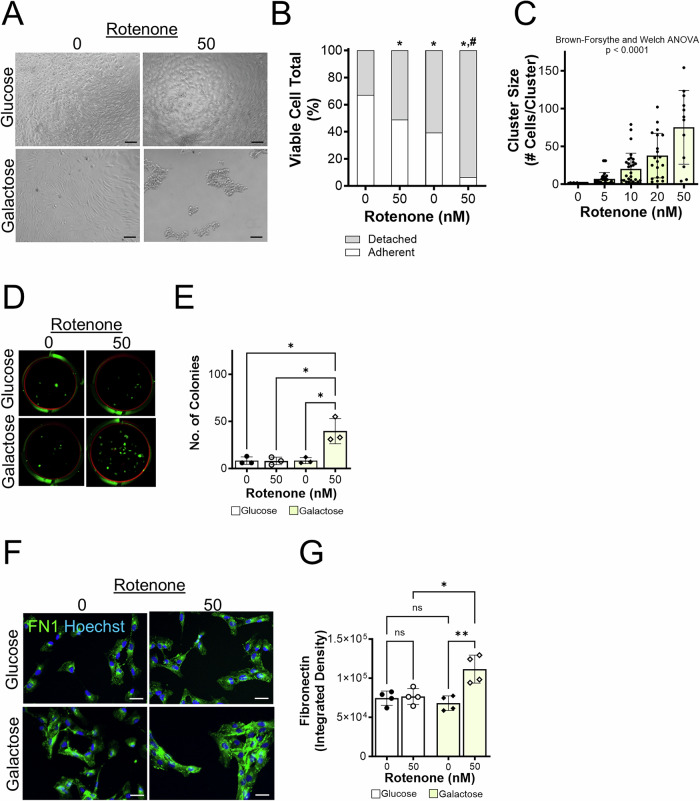
Glycolytic restriction reveals loss of attachment and clustering of BHPrS1 cells induced by mitochondrial ETC C1 dysfunction. **A** Representative images of cells grown in glucose or galactose, +/− 50 nM rotenone or DMSO vehicle control on Day 6. Scale bar, 200 µm. **B** Quantitation of detached and adherent BHPrS1 cells (trypan blue assay) 48 h after indicated growth conditions, *p < 0.05 vs. glucose conditions, #*p* < 0.05 vs. galactose conditions. **C** Quantitation of the effect of rotenone (0–50 nM) on cell cluster size in BHPrS1 cells grown in galactose. **D** Colony formation assay of detached cells isolated from cells grown in glucose or galactose, +/− 50 nM rotenone. Detached cells were isolated from the culture and re-seeded (1000 cells/well) in glucose containing media. After 6 days of growth, colonies were stained with methylene blue and dried and imaged. **E** Quantitation of the number of cell colonies measured using PhotoShop software. **F** Immunofluorescent staining of fibronectin (green) in cells treated with rotenone or DMSO vehicle control for 48 h. Scale bar, 50 µm. **G** Quantitation of immunostaining intensity. *p < 0.05; #*p* < 0.05 vs galactose; **p < 0.01; ***p < 0.001; ****p < 0.0001; ns non-significant.

SIPS induced in BHPrS1 cells by growth in galactose and rotenone treatment represented a phenotype of the subset of cells that remained attached to the culture plates at the termination of treatments. However, the more prominent effect of glycolytic restriction coupled with CI inhibition was the induced detachment of adherent BHPrS1 stromal cells following 48 h of rotenone treatment (50 nM). Phase contrast micrographs illustrated the impact of rotenone and glycolytic restriction on adherent cells pre-conditioned in glucose or galactose, then treated with or without rotenone for 48 h (Fig. 3A). The detached and adherent cell fractions were separately collected and their viability quantified using a trypan blue exclusion assay. Rotenone increased the detachment of viable cells, and this detachment was significantly increased by glycolytic restriction (Fig. 3B). In fact, detached cells obtained following glycolytic restriction and CI inhibition uniquely coalesced into large clusters (Fig. 3A, lower right panel). The size of the clusters was increased in a dose-dependent manner with increasing concentrations of rotenone (0–50 nM) under glycolytic restriction conditions (Fig. 3C). To further assess the viability of detached cells from each culture condition, colony formation assays were performed by re-seeding the same number of detached and dispersed cells/well (i.e., 1000 cells/well) into glucose-containing media for six days. Cell colonies were then stained with methylene blue and counted. Colony formation assays demonstrated a significant increase in the number of cells that were able to re-attach and form colonies established from the detached and dispersed cell clusters in rotenone coupled with glycolytic restriction compared to cells from glucose with or without rotenone or in galactose without rotenone (Fig. 3D, E).

The formation of detached cell clusters uniquely in BHPrS1 cells exposed to rotenone under glycolytic restriction enhanced their viability and is reminiscent of the phenotype of cells that are anoikis resistant. Some cells, including metastatic cancer cells [32, 33], upregulate fibronectin in response to detachment, which is critical for the formation of cell aggregates and contributes to anchorage-independent survival, or anoikis resistance [34]. IF staining of attached BHPrS1 cells demonstrated an increased expression of fibronectin induced by rotenone under glycolytic restriction conditions (Fig. 3F, G) providing a plausible mechanism for their enhanced formation into viable cell clusters under these conditions.

### ZC3H4 as a putative regulator of CI that is down-regulated in BPH

**Fig. 4:**
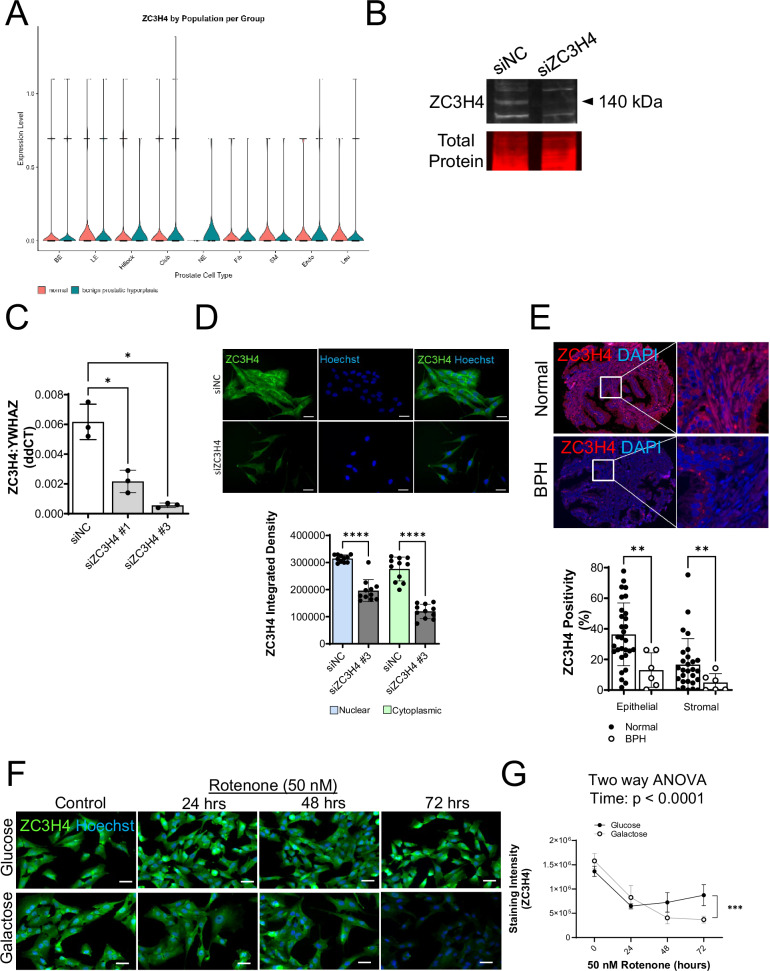
ZC3H4 expression is decreased in human BPH tissues and decreased by glycolytic restriction coupled with CI inhibition. **A** Violin plot of single cell RNA-Seq data showing decreased expression of ZC3H4 in BPH compared to Normal prostate in Hillock Epithelial and Smooth Muscle (SM) cells. BE basal epithelial, LE luminal epithelial, NE neuroendocrine, Fib fibroblast, SM smooth muscle, Endo endothelial, Leu leukocyte. **B** Western blotting analysis of ZC3H4 protein following siNC or siZC3H4 treatment in BHPrS1 cells and total protein staining of the same membrane. **C** ZC3H4 knockdown using two different siRNAs, siNC, non-coding control siRNA. **D** Upper panel. Immunofluorescent staining of ZC3H4 (green) in BHPrS1 cells treated with NC or ZC3H4 knockdown. Nuclei were stained with Hoechst (blue). Scale bar, 50 µm. Lower panel. Quantitation of ZC3H4 staining intensity. **E** Upper panel. Representative immunofluorescent images of ZC3H4 (red) and DAPI staining (blue) in histologically normal and BPH tissues from age-matched patients. Lower panel. Quantitation of epithelial Normal (*n* = 30) and BPH (*n* = 6) and stromal Normal (*n* = 28) and BPH (*n* = 6) ZC3H4 staining in human prostate tissues. Scale bar, 50 µm. **F** Immunofluorescent staining of ZC3H4 in BHPrS1 cells grown in either glucose or galactose with rotenone (50 nM) or DMSO vehicle control at indicated timepoints (i.e., 0, 24, 48 or 72 h rotenone). **G** Quantitation of ZC3H4 staining in BHPrS1 cells. *p < 0.05; **p < 0.01; ***p < 0.001; ****p < 0.0001; ns non-significant.

Fibroblasts in culture can acquire anoikis resistance under conditions of induced detachment, which may reflect changes accompanying tissue remodeling and fibrosis in intact tissue [35, 36]. The zinc finger transcription termination factor ZC3H4 [37, 38] was recently implicated in promoting anoikis resistance in lung fibroblasts, a maladaptation that leads to the development of a pro-fibrotic phenotype [35]. Interrogation of a scRNA-Seq data set obtained from normal and BPH tissues [39] revealed variable cell-specific changes in ZC3H4 mRNA levels (Fig. 4, Table 1). Notably, ZC3H4 expression decreased in hillock epithelial and smooth muscle cells of BPH tissue compared to normal prostate (Fig. 4A). Since ZC3H4 protein levels are distinctly regulated in other cell systems [37, 40–45], we evaluated ZC3H4 protein in normal and BPH tissue in a previously assembled prostate tissue microarray. The ZC3H4 antibody used for these studies was validated by examining Western blotting, mRNA and IF staining and in BHPrS1 cells with siRNA mediated ablation of ZC3H4 (Fig. 4B–D). Original western blot images listed in Supplementary Fig. S3. The validated ZC3H4 antibody revealed decreased accumulation of ZC3H4 protein in both the stromal and epithelial compartments of BPH specimens compared to normal tissues (Fig. 4E). Since mitochondrial function is reduced in BPH prostate tissues [1], we examined the impact of CI inhibition on ZC3H4 protein levels in vitro. Glycolytic restricted BHPrS1 cells treated with rotenone for 72 h led to a dramatic reduction in ZC3H4 protein (Fig. 4F, G). Thus, the in vitro effect of CI inhibition in human prostate stromal cell line BHPrS1 mirrored the effects on ZC3H4 protein accumulation in BPH tissue.

**Table 1:** ZC3H4 fold-change in mRNA expression in prostate cell types from BPH and Normal human prostate sc-RNA Seq dataset [18].

Cell Type	BPH:Normal
Basal epithelial	0.876249
Luminal epithelial	0.704811
Hillock	0.559926
Club	1.583987
Neuroendocrine	Inf
Fibroblast	0.857442
Smooth muscle	0.417989
Endothelial	1.210455
Leuokocyte	0.963311

**Fig. 5:**
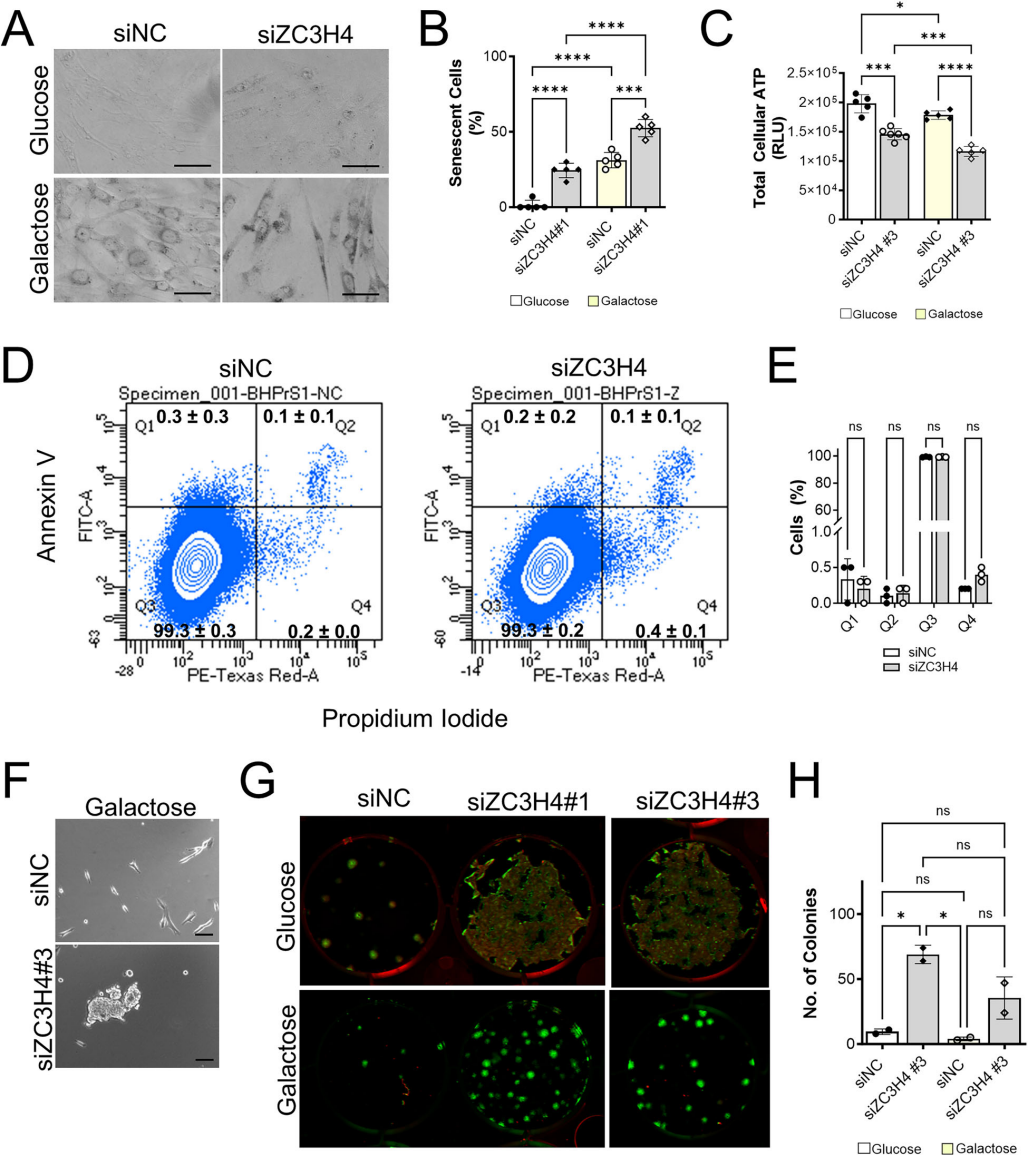
Potential role for ZC3H4 in senescence, attachment, adhesion and anoikis resistance. **A** Sudan Black B staining of lipofuscin in BHPrS1 cells treated with ZC3H4 knockdown (siZC3H4) or non-coding control siRNA (siNC) for 48 h. Scale bar, 50 µm. **B** Quantitation of the percentage of Sudan Black B positive senescent cells following treatment. **C** Impact of ZC3H4 knockdown on ATP levels in glucose or galactose conditions. **D** Representative flow cytometry chart of apoptotic (Q1), dead apoptotic (Q2), live (Q3) and dead (Q4) BHPrS1 cells following ZC3H4 knockdown. **E** Quantitation of apoptotic cells. **F** ZC3H4 knockdown induced detached aggregated cell clusters in BHPrS1 cells following trypsinization. Scale bar, 100 µm. **G** Colony formation assay of detached cells isolated from siZC3H4 knockdown or siNC control cells grown in glucose for 6 days. **H** Quantitation of the number of cell colonies measured using PhotoShop software. *p < 0.05, **p < 0.01; ***p < 0.001; ****p < 0.0001; ns, non-significant.

**Fig. 6:**
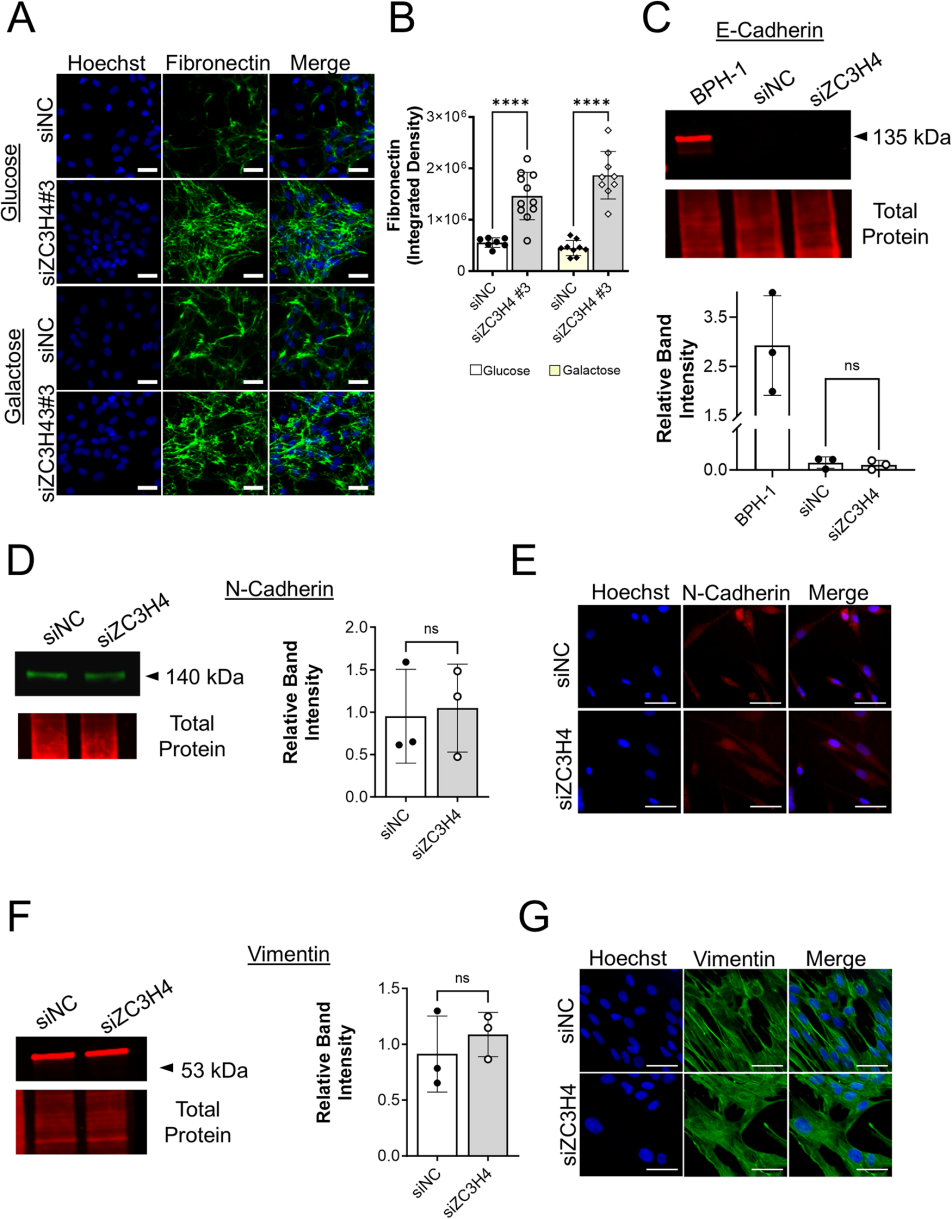
Potential role for ZC3H4 in anoikis resistance and mesenchymal-epithelial transition. **A** Impact of ZC3H4 knockdown on fibronectin immunofluorescent staining (green) in BHPrS1 prostate stromal cells. Nuclei were stained with Hoechst (blue). Scale bar, 50 µm. **B** Quantitation of fibronectin staining intensity in treated cells. **C** Western blotting and quantitative data of E-cadherin in BPH-1 prostate epithelial cells and siNC and siZC3H4 BHPrS1 cells relative to total protein (Supplementary Fig. S3). **D** Western blotting and quantitative data of N-Cadherin in BHPrS1 cells. **E** Representative immunofluorescent images of N-cadherin (red) and Hoechst staining (blue) in BHPrS1 cells treated with siNC or siZC3H4 knockdown in glycolytic conditions. **F** Western blotting and quantitative data of vimentin in BHPrS1 cells. **G** Representative immunofluorescent images of vimentin (green) and Hoechst staining (blue) in BHPrS1 cells treated with siNC or siBHPrS1 knockdown. Scale bar, 50 µm. ****p < 0.0001; ns non-significant.

Acute ZC3H4 ablation in the colorectal carcinoma cell line HCT116 and in HeLa cells led to decreased proliferation and elevated mRNA expression of genes associated with inflammation, stromal remodeling and metabolism [37], along with several pathways that are also impacted by mitochondrial dysfunction and known to play a role in BPH development and progression (Table 2). Thus, we sought to test whether ZC3H4 ablation could contribute to the phenotypes observed upon CI inhibition combined with glycolytic restriction in BHPrS1 cells. Knockdown of ZC3H4 in BHPrS1 cells significantly increased the number of SBB-positive cells and decreased ATP levels under both glycolytic and glycolytic restriction conditions (Fig. 5A–C) without inducing apoptosis (Fig. 5D, E). ZC3H4 knockdown also induced cell detachment and a clustering phenotype, particularly under glycolytic restriction conditions (Fig. 5F). Colony formation assays showed that detached siZC3H4 cells from both glucose or galactose conditions were viable and capable of forming colonies (Fig. 5G, H). Knockdown of ZC3H4 significantly induced an increase in fibronectin (Fig. 6A, B), but did not alter the expression of other epithelial to mesenchymal transition markers E-cadherin, N-cadherin or vimentin (Fig. 6D–G).

**Table 2:** Pathways modulated by ZC3H4 knockdown in HCT116 and HeLa cells [37].

Cell line	KEGG Pathway	Count	*p*-Value	Fold Enrichment
**HCT116**	TGF-beta signaling pathway	10	1.27E-04	5.14
	ECM-receptor interaction	7	8.91E-03	3.88
	Hippo signaling pathway	12	3.44E-04	3.73
	Protein digestion and absorption	7	1.83E-02	3.32
	Focal adhesion	12	2.61E-03	2.92
	Relaxin signaling pathway	7	4.77E-02	2.65
	MAPK signaling pathway	15	2.63E-03	2.49
**HeLa**	Metabolic pathways	30	5.61E-03	1.63
	Nicotinate and nicotinamide metabolism	4	9.27E-03	9.07
	Ubiquitin mediated proteolysis	5	8.68E-02	2.96

**Fig. 7:**
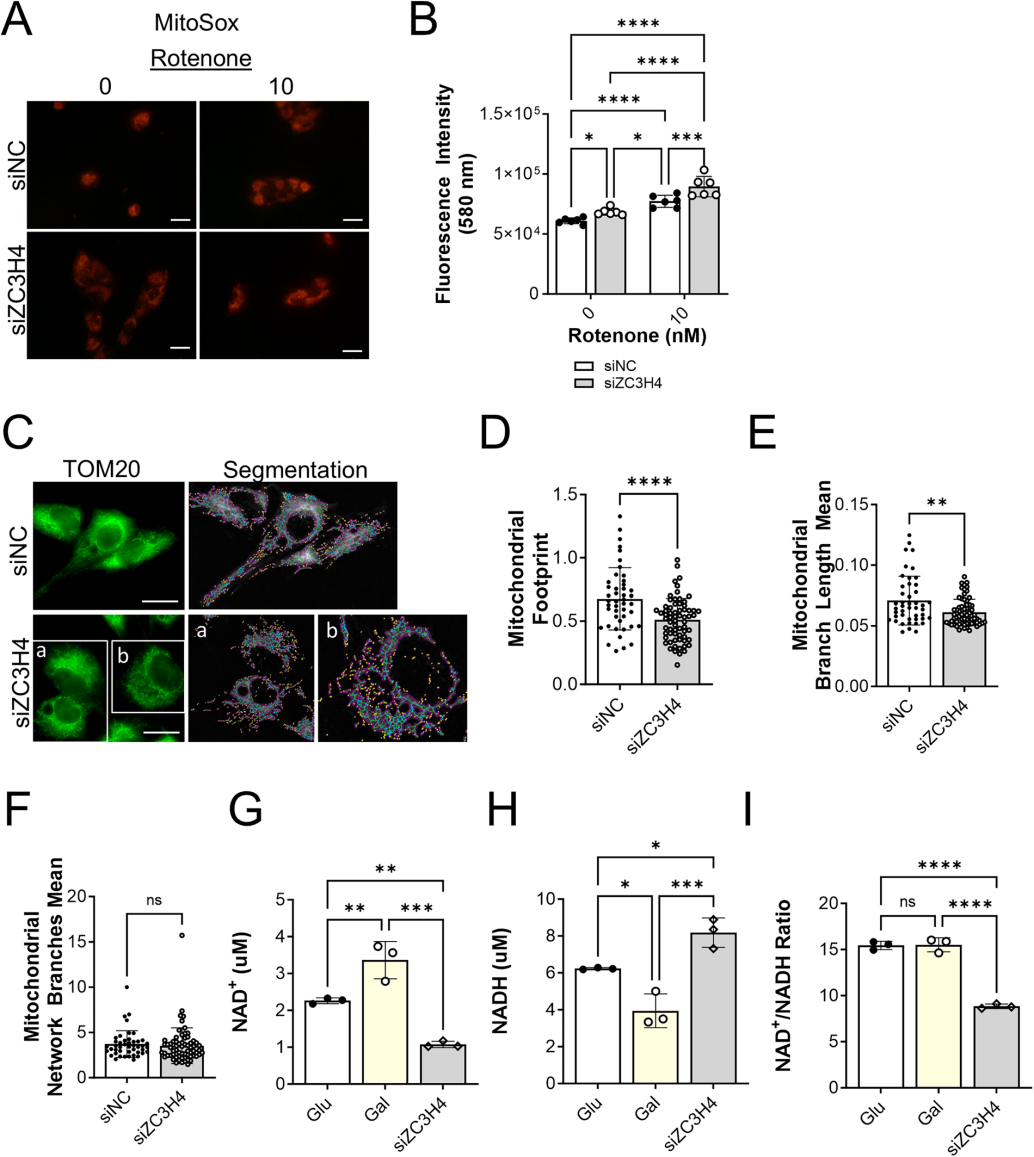
ZC3H4 knockdown induces mtROS, alters mitochondrial morphology and the NAD^+^/NADH ratio. **A** Impact of ZC3H4 knockdown on MitoSOX staining (red) of mitochondrial reactive oxygen species in BHPrS1 cells. Scale bar, 50 µm. **B** MitoSOX staining intensity was quantitated via microplate reader measurement at 580 nm. **C** TOM20 staining of mitochondria in BHPrS1 cells and mitochondrial segment analysis using MiNA. Branches are delineated in green, branch points in blue and branch endpoints as yellow dots. Scale bar, 10 µm. **D** Mitochondrial footprint, **E** Branch length and **F**. Network branches (data represent mean). **G** NAD^+^ levels in BHPrS1 cells grown in glucose, galactose and siZC3H4 treated cells in galactose conditions. **H** NADH levels. **I** NAD^+^/NADH ratio. *p < 0.05; **p < 0.01; ***p < 0.001; ****p < 0.0001; ns non-significant.

Mitochondrial ROS (mtROS) was measured using MitoSOX staining. BHPrS1 cells treated with rotenone (10 nM) had significantly increased levels of MitoSOX staining intensity (Supplementary Fig. S5). Similarly, ZC3H4 knockdown significantly increased mtROS levels and increased the sensitivity of BHPrS1 cells to low levels of rotenone (10 nM) (Fig. 7A,B). Mitochondrial fragmentation was also increased by ZC3H4 knockdown (Fig. 7C–F). Similar to the impact of glycolytic restriction in skeletal muscle cells [46], NAD^+^ levels were increased under glycolytic restriction conditions while NADH levels were decreased without significantly altering the NAD^+^/NADH ratio (Fig. 7G–I). Interestingly, ZC3H4 knockdown in glycolytic restriction conditions significantly reduced NAD^+^ levels and increased NADH levels compared to both glucose and galactose conditions, resulting in a significant reduction in the NAD^+^/NADH ratio (Fig. 7G–I). These results are similar to the effects on NAD^+^ and NAD^+^/NADH ratio observed by others in fibroblasts treated with rotenone [47, 48].

**Fig. 8:**
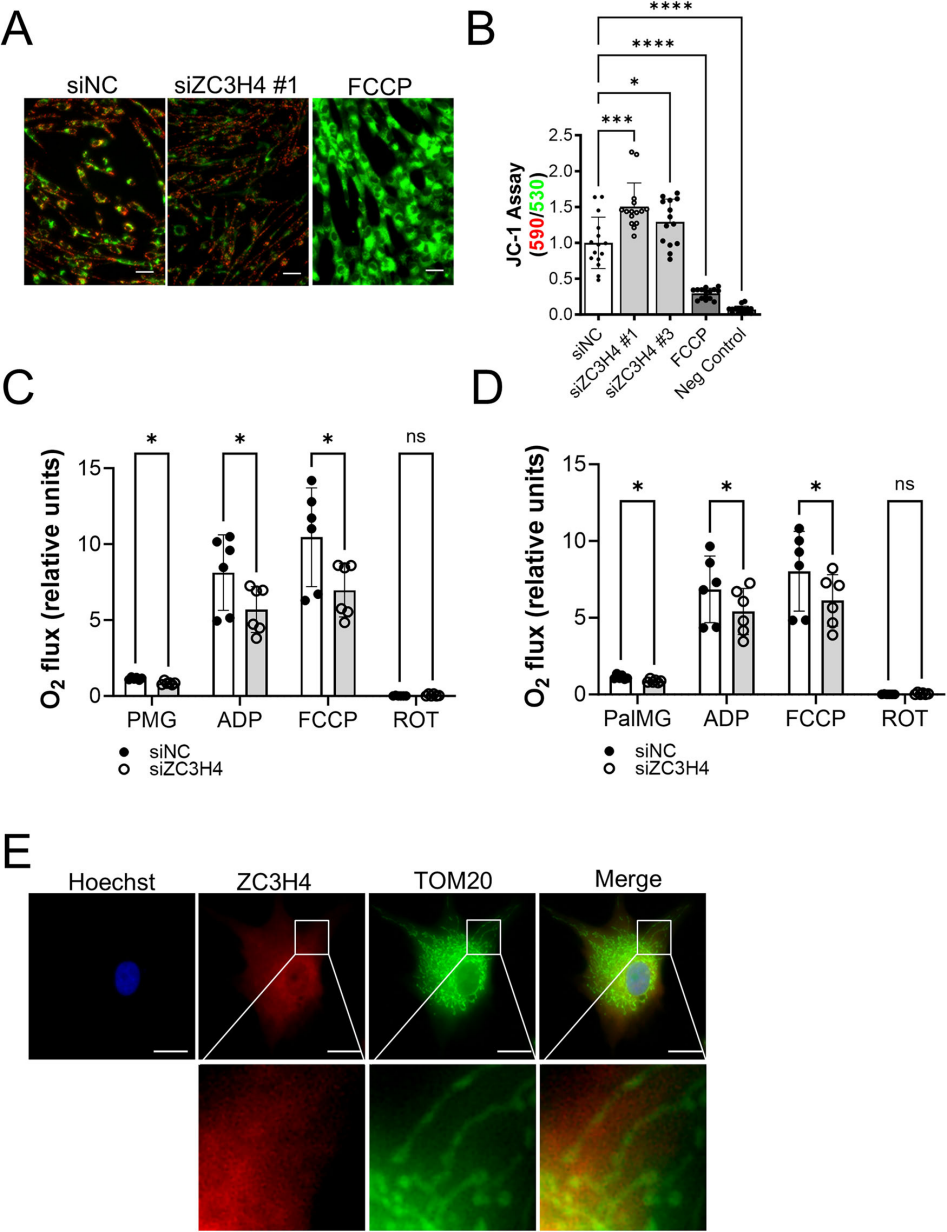
ZC3H4 regulation of mitochondrial function. **A** Mitochondrial membrane potential measurement via JC-1 fluorescent microplate assay in BHPrS1 cells following siZC3H4 knockdown or siNC control in galactose. **B** Quantitation of red to green fluorescence ratio. FCCP (100 µM) served as uncoupler, cells with no dye served as negative control. Scale bar, 50 µm. **C** Mitochondrial respiration in BHPrS1 cells was evaluated following ZC3H4 ablation (siZC3H4) compared to non-coding control (siNC). Mitochondrial basal or leak respiration was assessed in the presence of PMG (pyruvate, malate, glutamate) or **D** PalMG (palmitoyl-carnitine, malate, glutamate) and following addition of ADP to stimulate oxidative phosphorylation, FCCP to assess maximum electron transport chain activity and rotenone to inhibit complex I. Data were normalized each day to the average basal respiration for all samples on that given day. **E** Immunofluorescent staining of ZC3H4 (red) and TOM20 (green) in BHPrS1 cells. Scale bar, 10 µm. *p < 0.05; **p < 0.01; ***p < 0.001; ****p < 0.0001; ns non-significant.

Assessment of mitochondrial function following knockdown of ZC3H4 was also performed. Mitochondrial membrane potential was significantly increased by ZC3H4 knockdown under glycolytic restriction conditions suggesting that ZC3H4 ablation increased mitochondrial polarization (Fig. 8A, B). Mitochondrial respiratory capacity assessed in the presence of NADH-linked or Complex I substrates pyruvate and palmitoyl-carnitine was significantly reduced by ZC3H4 ablation (Fig. 8C, D). Co-staining of BHPrS1 cells with ZC3H4 and mitochondria marker TOM20 demonstrated two distinct staining patterns. BHPrS1 cells showed diffuse nuclear and cytosolic staining of ZC3H4 (red), while TOM20 showed intense staining (green) of the mitochondria (Fig. 8E). Query of ZC3H4 in the database repository the Integrated Mitochondrial Protein Index (IMPI) [49] identified ZC3H4 as predicted to not be associated or localized to mitochondrion. ZC3H4 was also not identified as a mitochondrial-associated protein in database repositories MitoCarta 3.0 [50] and the Human Protein Atlas [51]. RNA-Seq analysis identified the read-through enhancement of Complex I subunit genes following siRNA knockdown of ZC3H4 in the cell line HCT116 [37]. Complex I subunit genes NDUFAF3, NDUFA3, NDUFS3 had a log2 fold-change greater than 1, while NDUFB3 and NDUFA12 had a log2 fold-change less than −1 (Supplementary Fig. S6). ZC3H4 may therefore influence Complex I function through translation of a number of nuclear encoded Complex I subunit mRNAs. Taken together, these findings suggest that ZC3H4 knockdown phenocopies the effects of rotenone inhibition of CI and mitochondrial integrity in prostate stromal cells, implicating ZC3H4 as a novel regulator of mitochondrial function.

## Discussion

What are the consequences of the age-dependent deterioration of mitochondrial CI function, which we previously observed in BPH tissue [1]? In this study we focused on the impact of mitochondrial CI inhibition of prostate stromal cells and find that it induces SIPS and stromal cell remodeling, as reflected most prominently in altered attachment and adhesion properties as well as anoikis resistance in the BHPrS1 cell line. Glycolytic restriction amplifies the impact of CI inhibition on BHPrS1 cells to induce these phenotypes as cells become more reliant on OXPHOS for efficient ATP production and metabolic stability. The robust recovery of BHPrS1 cell growth and overall ATP production upon direct entry of glucose into glycolysis suggests that glycolytic byproducts allow for metabolic flexibility within mitochondria to provide the necessary energetic requirements for efficient cell growth in prostate stromal cells. This is also supported by the relative insensitivity of BHPrS1 cells to CI inhibition by rotenone when supplied with glucose as a 6-carbon source. Although glycolytic restriction coupled with CI inhibition did not induce cell cycle arrest, the increased cell size and granularity, the accumulation of lipofuscin, as well as an increase in IL-6 mRNA support the induction of a SIPS phenotype. We also observed an increased expression of fibronectin in CI inhibited BHPrS1 cells, which has been associated with SIPS in other fibroblastic cell lines and may contribute to morphological changes in senescent cells [31, 52, 53]. The relevance of our in vitro results to pathological changes in the prostate of BPH patients is supported by the increase in senescent prostate stromal cells and SASP-associated cytokines in BPH [54]. Increased fibronectin deposition has also been observed in BPH tissues [55] and in response to *E.coli* induced prostatic inflammation in rat models of BPH [56].

A surprising feature of altered adhesion (i.e., cell-cell and cell-substratum) of BHPrS1 cells in response to CI inhibition is the pronounced accumulation of viable cell clusters, reminiscent of anoikis resistance. This phenotype led us to uncover a potential role for the anoikis-resistance protein ZC3H4 in the maintenance of benign human prostate stromal cell homeostasis. We show that ZC3H4 expression is decreased in the stromal compartment of BPH patient prostates and phenocopies BHPrS1 cell phenotypes elicited by CI inhibition under conditions of glycolytic restriction, thus identifying ZC3H4 as a novel regulator of CI function. ZC3H4 may play a role in lung fibrosis and inflammation [35] and its ablation in the colorectal carcinoma cell line HCT116 led to decreased proliferation and altered expression of genes associated with inflammation, fibrosis and cell adhesion [37], several pathways which are known to play a role in BPH development and progression, including TGF-β, ECM interactions and focal adhesion. In the prostate, loss of stromal ZC3H4-regulated mitochondrial function may trigger an anoikis resistance response that contributes to remodeling of the substratum and changes in epithelial proliferation and immune cell infiltration characteristic of BPH.

While ZC3H4 is required for early embryogenesis in mice [43], its role in human physiology and pathology has only been established in murine and cell-line models of lung fibrosis, in this case acting distinctly within different cell types [35, 40–42, 45]. For example, in a SiO2-induced mouse model of lung fibrosis, ZC3H4 expressed in macrophages and monocytes regulated the progression of inflammation [42, 45]. Alternatively, in lung epithelial cells, ZC3H4 promoted EMT while it contributed to fibroblast activation by multiple pathways [35, 40–42, 45]. Thus, as in the lung, ZC3H4 may be a multi-modal regulator of prostate pathology and pathophysiology acting in multiple cell types when its expression is limited to promote distinct features of LUTS/BPH (i.e., fibrosis, inflammation, senescence). ZC3H4 expression in multiple cell types in the prostate support this contention, although curiously only a few cell types show altered ZC3H4 mRNA expression in BPH as assessed by scRNAseq including the most abundant stromal cell type, smooth muscle. However, posttranscriptional regulation of ZC3H4 by a specific circular RNA (ZC3H4 circRNA) [44] may impact changes in ZC3H4 protein expression, which are robust in both the stromal and epithelial compartment of BPH relative to normal prostate. circRNAs play essential roles in various pathophysiological conditions via interactions with numerous molecules at different levels, including acting as miRNA sponges or decoys, protein interactions, transcription termination, and mediating cell-cell communications [57]. They have also been shown to play a role in aging and fibrosis [58]. High throughput screening identified elevated circZC3H4 levels in response to SiO_2_-induced pulmonary inflammation [45]. Although down-regulation of ZC3H4 in BPH smooth muscle cells was identified via scRNA-Seq, alterations in ZC3H4 protein expression by profibrotic or other triggers could also operate through circZC3H4 regulation of ZC3H4 mRNA translation and may be associated with changes in ZC3H4 protein in BPH tissue with distinct pathologies.

The precise molecular function attributed to ZC3H4 that promotes these pathologies is not known. A number of recent genome-wide studies have uncovered a function for ZC3H4 in regulating the termination (and splicing) of extragenic transcription including promoter anti-sense RNA (pasRNA), enhancer long noncoding RNA (elncRNA) and super enhancer RNA (seRNA) [37, 59]. In addition, ZC3H4 regulates the transcription termination of a wide variety of coding and non-coding RNAs within pathways involved in EMT, fibrosis and metabolism [37, 38, 59, 60]. Irrespective of whether a novel uncovered function of ZC3H4 or its impact on select coding or non-coding transcripts is responsible for its role in maintaining prostate stromal homeostasis, the fact that it phenocopies the effects of CI inhibition under glycolytic restricted conditions implies that one or more of its activities is linked to maintaining mitochondrial function and could be important for promoting metabolic flexibility in any cell type in which it is expressed (i.e., including prostate epithelial cells). The stromal compartment is a critical regulator of prostate homeostasis. Stromal cells in BPH tissues have been characterized by altered mitochondrial function [1], chemokine and cytokine expression [54, 61], increased inflammation [62–65], extracellular matrix remodeling factors and fibrosis [54, 66–68]. Therefore, the phenotypes observed in ZC3H4 knockdown of BHPrS1 cells, including increased fibronectin, altered mitochondrial function and anoikis-resistance, could all contribute to stromal remodeling and fibrosis in the aging prostate. The multiple phenotypes observed in BHPrS1 cells induced by ZC3H4 knockdown may also be observed in multiple cell types located within the prostate stromal compartment. Furthermore, some BPH phenotypes observed in prostate epithelial cells may be sensitive to impairment of CI function triggered by reduced ZC3H4 expression, even though their energetic needs are mainly provided by glycolysis [6]. For example, senescence of epithelial and stromal cells and elevated SASP chemokines have been reported in BPH tissue [54, 69], supporting the translational relevance of the senescence induced by ZC3H4 loss in BHPrS1 cells. Alteration of mitochondrial markers in both the stromal and epithelial compartments of BPH tissues [1] could also be related to ZC3H4 loss.

In summary, we have identified a novel regulator of prostate stromal cell homeostasis, ZC3H4, which may promote mitochondrial function and thereby limit pathophysiologic changes in this compartment (i.e., SIPS, anoikis resistance) that are features of the aging prostate. Given its widespread expression and the involvement of “spurious intragenic transcription” in aging and senescence [70], the transcription termination function of ZC3H4 [37, 38] could be important for limiting mitochondrial dysfunction in many other diseases and conditions associated with unhealthy aging.

## Supplementary information

Supplementary Information

Original Data

## Data Availability

All data generated and analyzed during the current study are available from the corresponding author, Laura E. Pascal, upon reasonable request.
