# Supplementary material for: ZC3H4, a novel regulator of mitochondrial complex I, impacts prostate stromal cell senescence, attachment, adhesion and anoikis resistance
Source: Cell Death Dis. 2025 Oct 21;16(1):741. doi: 10.1038/s41419-025-08027-8 (PMC12540758; doi:10.1038/s41419-025-08027-8)
Supplement: Supplementary file 1 — Supplementary Information [file 41419_2025_8027_MOESM1_ESM.pdf]

SUPPLEMENTARY INFORMATION

ZC3H4, a Novel Regulator of Mitochondrial Complex I, Impacts Prostate Stromal Cell Senescence, Attachment, Adhesion and Anoikis Resistance

Teresa T. Liu[1], Mia J. Carrarini[2,3], Livianna K. Myklebust[2,4], Kegan O. Skalitzky[1,2], Nathalie El-Khoury[2], Ian J. Sipula[5], Alexander Chang[6], Vishal Soman[6], Ailing Liu[7], Nnamdi Ihejirika[2], Uma R. Chandran[6], Michael J. Jurczak[5], William A. Ricke[1], Donald B. DeFranco[2,8], and Laura E. Pascal[2,9]*

[1]Department of Urology, University of Wisconsin, Madison, WI, USA
[2]Department of Pharmacology and Chemical Biology, University of Pittsburgh School of Medicine, Pittsburgh, PA, USA
[3]Department of Biological Sciences, Carnegie Mellon University, Pittsburgh, PA, USA
[4]Molecular Genetics and Developmental Biology Graduate Program, University of Pittsburgh School of Medicine, Pittsburgh, PA, USA
[5]Department of Medicine, Division of Endocrinology and Metabolism, University of Pittsburgh School of Medicine, Pittsburgh, PA, USA
[6]Department of Biomedical Informatics, University of Pittsburgh, Pittsburgh, PA, USA
[7]Department of Immunology, University of Pittsburgh School of Medicine, Pittsburgh, PA, USA
[8]Pittsburgh Institute for Neurodegenerative Diseases, University of Pittsburgh School of Medicine, Pittsburgh, PA, USA
[9]UPMC Hillman Cancer Center, University of Pittsburgh School of Medicine, Pittsburgh, PA, USA

*Correspondence to: Laura E. Pascal, email: lep44@pitt.edu

This PDF file includes:

Tables S1, S2

Figures S1 to S5

Supplemental Table S1. Primer sequences for qRT-PCR

Gene	HUGO* Gene Name	Forward	Reverse
FN1	Fibronectin 1	CAGGATCACTTACGGAGAAACAG	GCCAGTGACAGCATACACAGTG
IL6	Interleukin 6	CCTGAGAAAGGAGACATGTAACAAGA	GGAAGGTTCAGGTTGTTTTCTGC
IL1b	Interleukin 1 beta	TGGCAGAAGTACCTGAGCTCGC	GCCGCCATCCAGAGGGCAGA
FN1	Fibronectin 1	CAGGATCACTTACGGAGAAACAG	GCCAGTGACAGCATACACAGTG
GAPDH	Glyceraldehyde-3-phosphate dehydrogenase	GGAGCGAGATCCCTCCAAAAT	GGCTGTTGTCATACTTCTCATGG
TNF	Tumor necrosis factor	GTGATCGGCCCCCAGAGGGAA	TGGAGCTGCCCCTCAGCTTGA
YWHAZ	Tyrosine 3-monooxygenase/tryptophan 5-monooxygenase activation protein zeta	TGATCCCCAATGCTTCACAAG	GCCAAGGTAACGGTAGTAATC
ZC3H4	Zinc finger CCCH-type containing 4	GAATGATGCCCCCTATCCCG	CTTCGTAGTGCCCGTAGTCC

*HUGO Gene Nomenclature Committee at the European Bioinformatics Institute www.genenames.org

Supplemental Table S2. List of Primary Antibodies

Antibody	Source	Clone, Catalog #	RRID	Species raised in, Monoclonal or Polyclonal	Application, Dilution
E-cadherin	Cell Signaling Technology	24E10, 3195	AB_2291471	Rabbit, monoclonal	WB, 1:1,000
Fibronectin 1	Cell Signaling Technology	E5H6X, 26836	AB_2924220	Rabbit, monoclonal	IF, 1:1,000
N-cadherin	Cell Signaling Technology	13A9, 14215	AB_2798427	Mouse, monoclonal	IF, 1:100<br>WB, 1:1,000
Vimentin	Bioss Antibodies	Bs-0756R	AB_10855343	Rabbit, polyclonal	IF, 1:100<br>WB, 1:500
PAI-1	Abcam	Ab66705	AB_1310540	Rabbit, polyclonal	IF, 1:500
TOM20	Santa Cruz Biotechnology	F10, sc-17764	AB_628381	Mouse, monoclonal	IF, 1:10,000
ZC3H4	Proteintech	20041-1-AP	AB_10667456	Rabbit, polyclonal	IF, 1:500<br>WB, 1:250

RRID, Research Resource Identification; WB, western blotting; IF, immunofluorescent staining

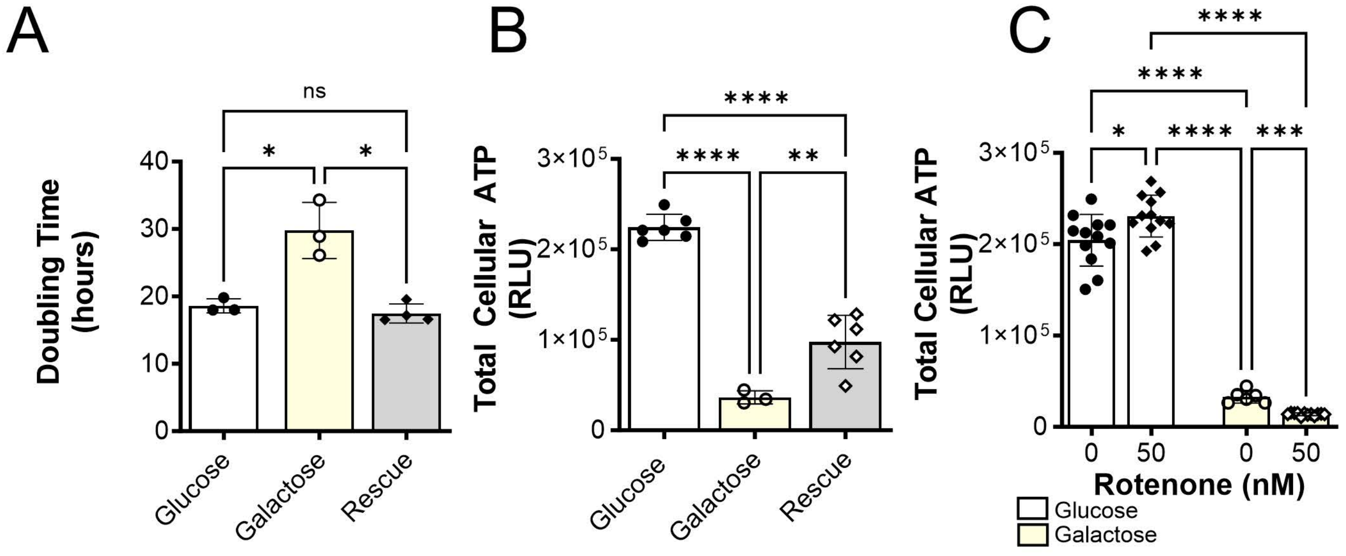

Supplemental Figure S1. A. Restoration of cell doubling time in BHPrS1 cells following return to glycolytic conditions (rescue) on Day 4, doubling time calculated for cells collected on Day 7, after 3 days in rescue conditions. **B**. Quantitation of ATP in TP18S317 patient-derived BPH stromal cells on Day 6 in glucose, galactose or rescue conditions via CellTiter luminescence assay. **C**. Quantitation of ATP in TP18S317 patient-derived BPH stromal cells on Day 6 in glucose or galactose +/- rotenone (50 nM) culture conditions via CellTiter luminescence assay. *, $p < 0.05$; **, $p < 0.01$, ***, $p < 0.001$; ****, $p < 0.0001$, ns, non-significant.

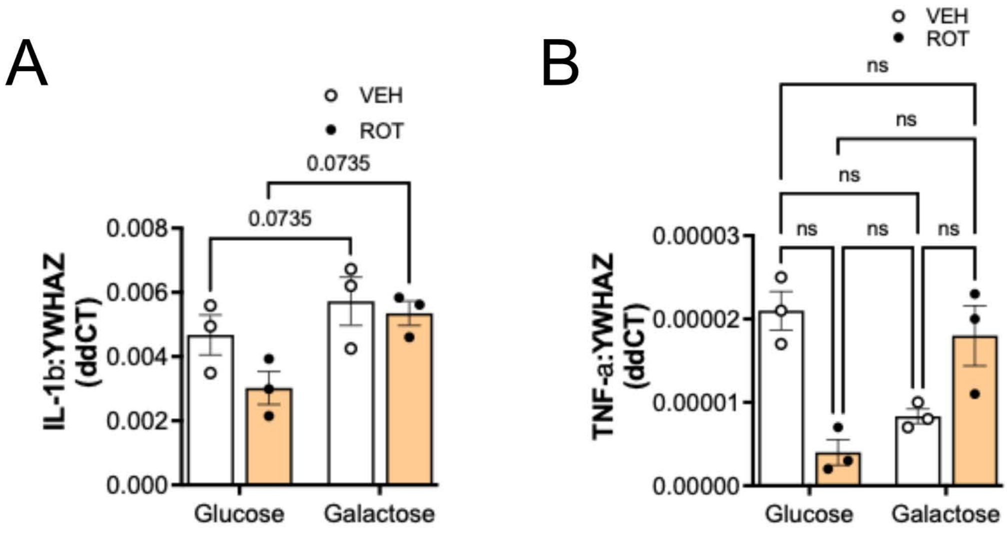

Supplemental Figure S2. Impact of glycolytic restriction and rotenone on the expression of SASP cytokines **A**. IL-1β and **B**. TNF-α as determined by qRT-PCR on Day 5. *, $p < 0.05$; **, $p < 0.01$; ***, $p < 0.001$; ****, $p < 0.0001$. Experiment was repeated 3 times.

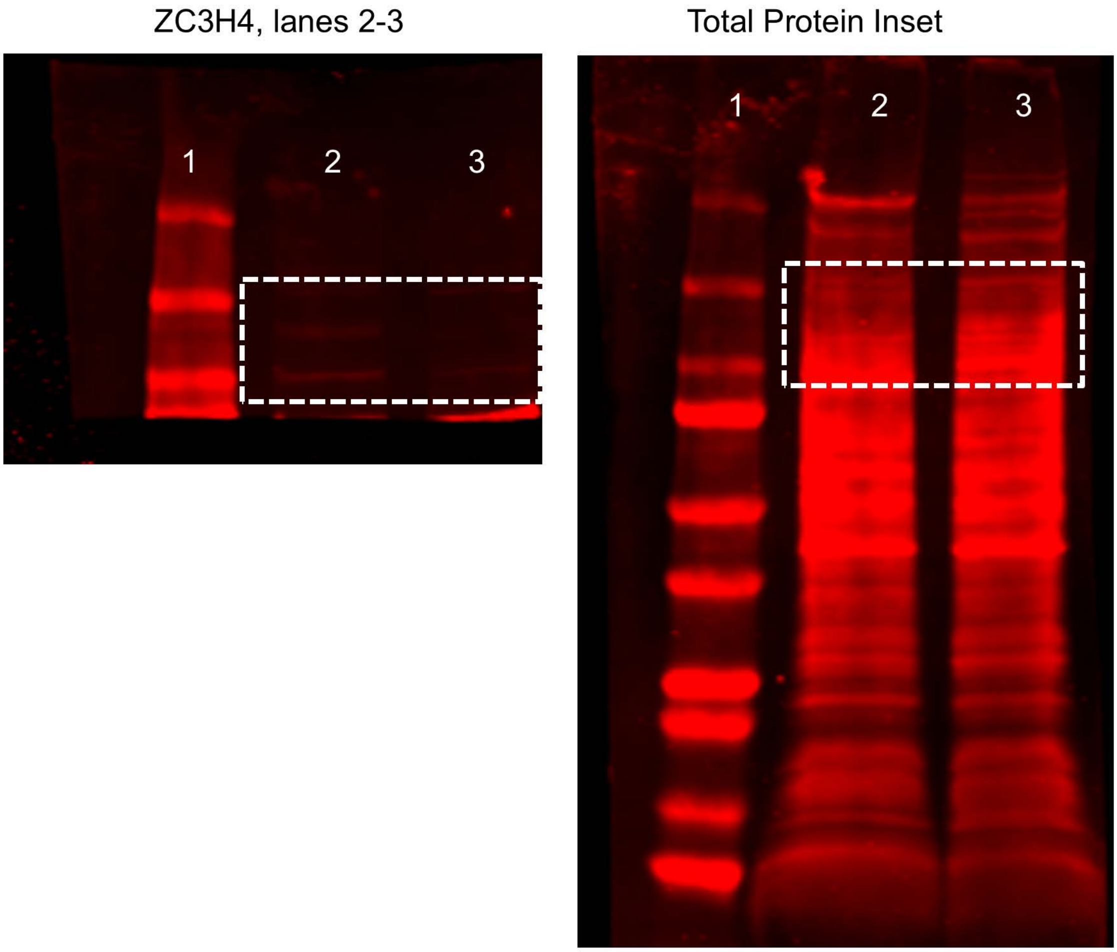

Supplemental Figure S3. Original western blot images for Figure 4B. BHPrS1 cells separated onto PVDF membrane and probed with ZC3H4 antibody (left panel, lanes 2-3). Total protein (right panel, lanes 2-3). Protein ladder in lane 1.

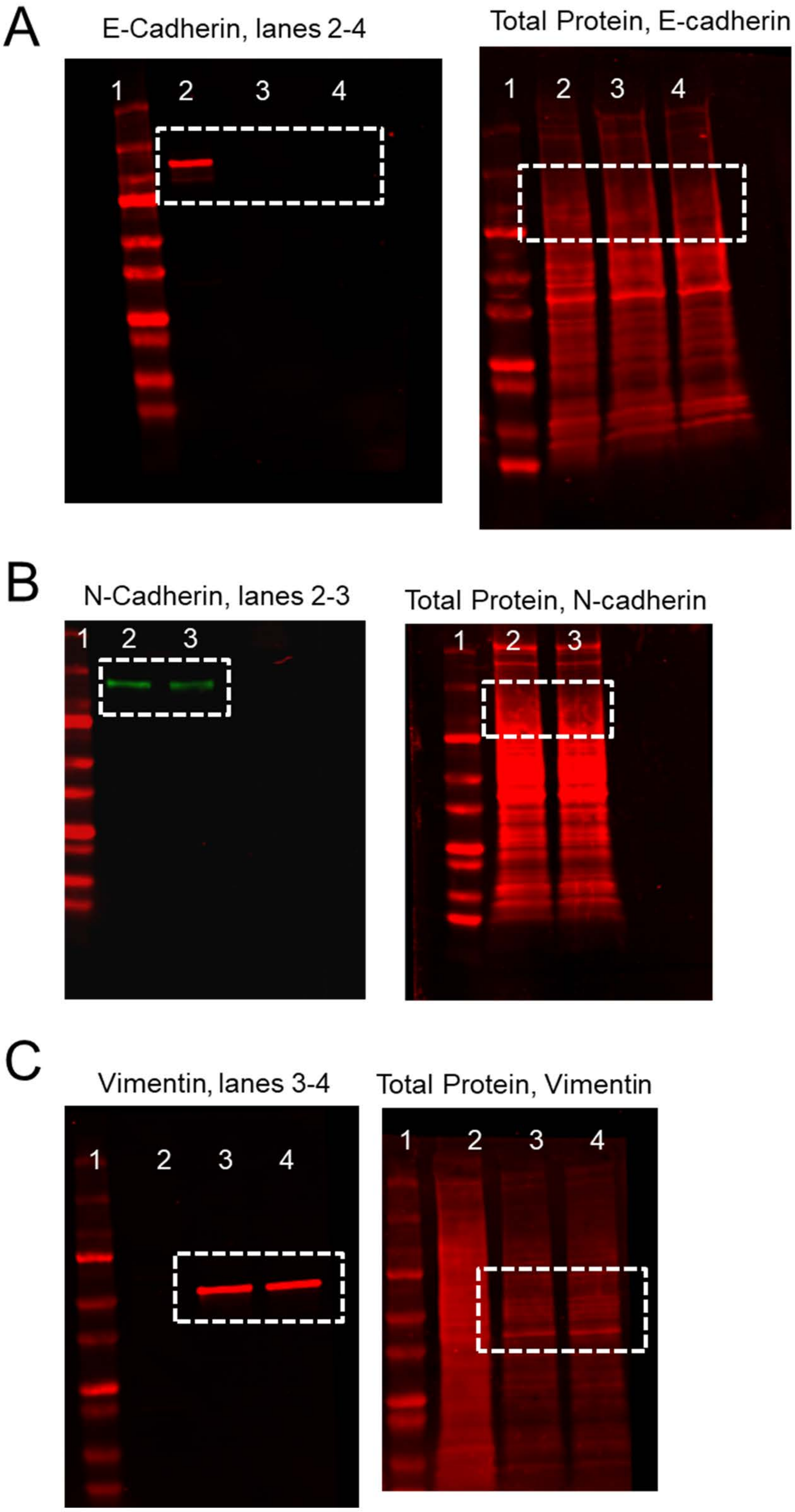

Supplemental Figure S4. Original western blot images for Figure 6. BHPrS1 cells separated onto PVDF membrane and probed with: **A**. E-cadherin antibody (left panel, lanes 2-4). Total protein (right panel, lanes 2-4). Protein ladder in lane 1. **B**. N-cadherin (left panel, lanes 2-3). Total protein (right panel, lanes 2-3). **C**. Vimentin (left panel, lanes 3-4). Total protein (right panel, lanes 3-4).

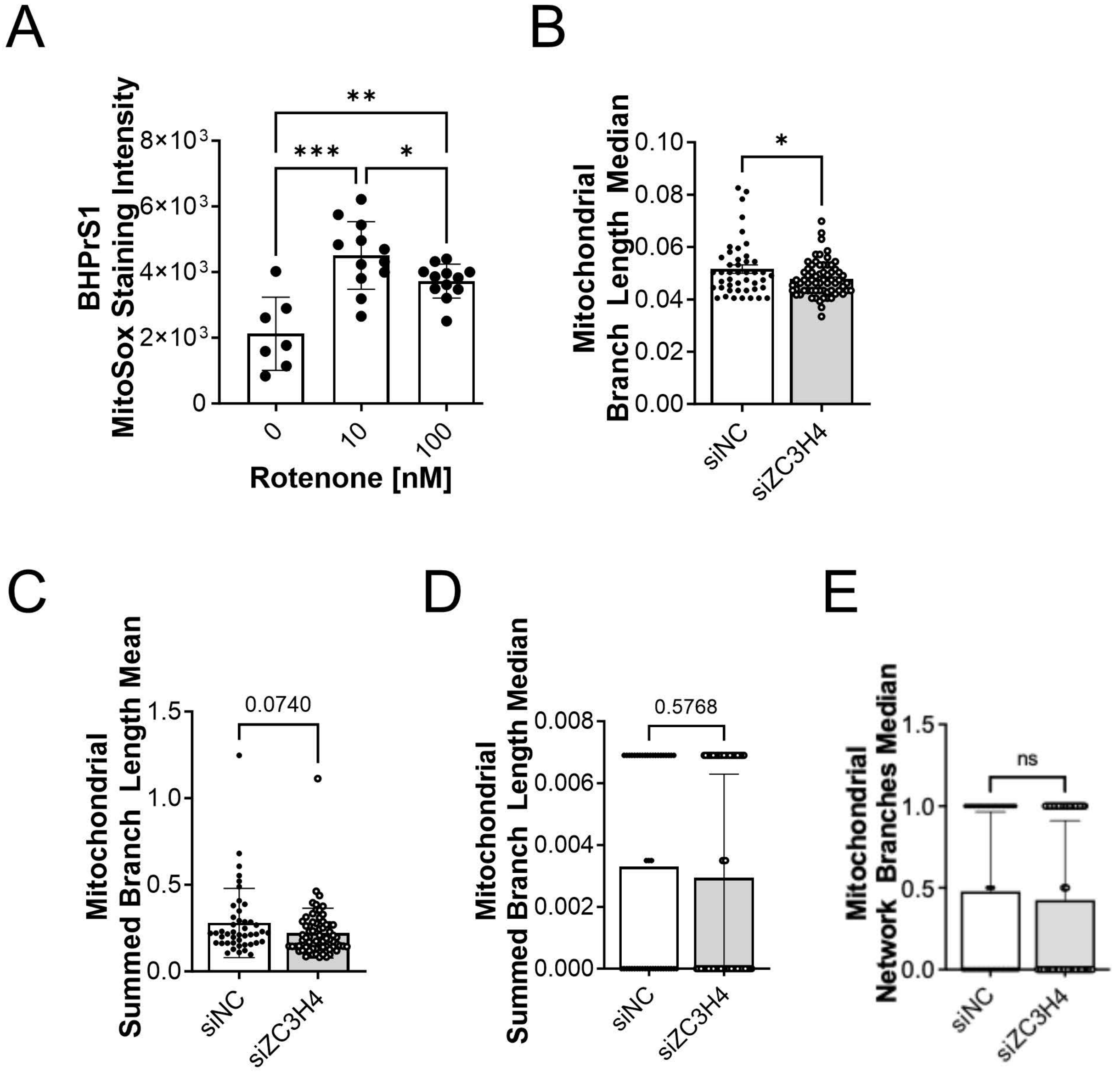

Supplemental Figure S5. **A**. MitoSOX staining intensity in BHPrS1 cells treated with 0, 10 or 50 nM rotenone for 3 h. **B**. Median mitochondrial branch length in BHPrS1 cells treated with non-coding control si (siNC) or ZC3H4 pooled si (siZC3H4). **C**. Median summed mitochondrial branch length. **D**. Mean summed mitochondrial branch length. **E**. Median mitochondrial branch network. *, p<0.05; **, p<0.01; ***, p<0.001; ns, non-significant.

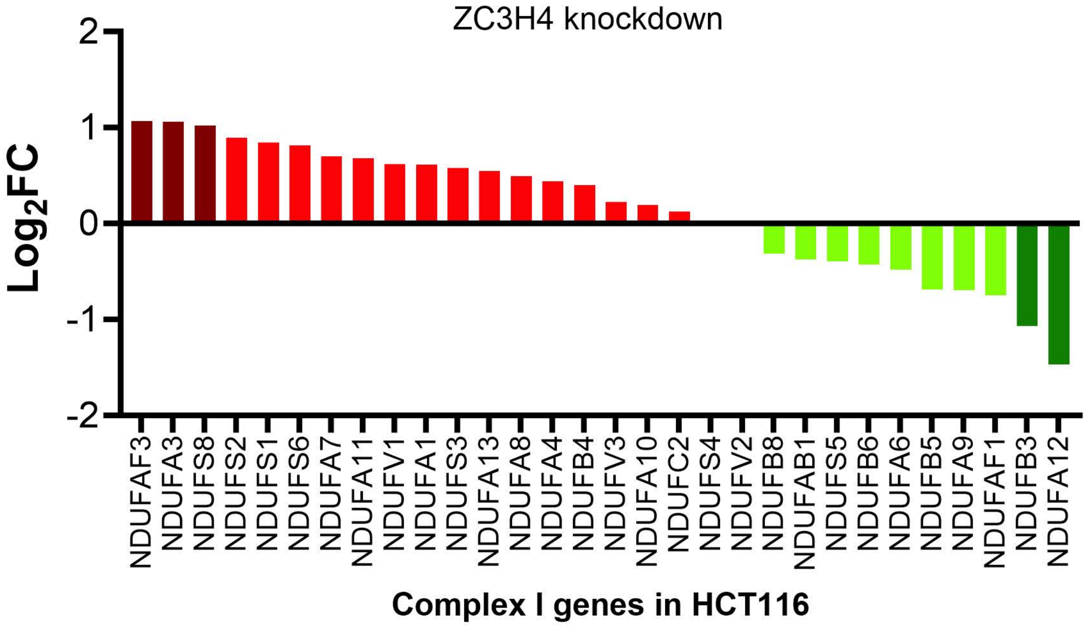

Supplemental Figure S6. Graph showing the expression of nuclear encoded Complex I subunit genes following ZC3H4 depletion in HCT116 cells 37. Read-through enhancement is shown on a log2 fold scale, scored as a ratio of reads upstream (-500 bp) and downstream (+1 kb) of the polyadenylation signal and results are shown as read-through enhancement (red) or decrease (green) on a log2 fold scale. Genes with log2 fold change (Log2FC) of 1 or more are identified with darker red bars while genes with log2 fold of -1 or less are identified with darker green bars.
