# Supplementary material for: ZC3H4, a novel regulator of mitochondrial complex I, impacts prostate stromal cell senescence, attachment, adhesion and anoikis resistance
Source: Cell Death Dis. 2025 Oct 21;16(1):741. doi: 10.1038/s41419-025-08027-8 (PMC12540758; doi:10.1038/s41419-025-08027-8)
Supplement: Supplementary file 2 — Original Data [file 41419_2025_8027_MOESM2_ESM.pdf]

Original data: ZC3H4 Western blot from Figure 4 and Supplemental Figure S3

ZC3H4 original western blot:
Protein ladder lane 1
siNC BHPrS1 lane 2
siZC3H4 BHPrS1 lane 3

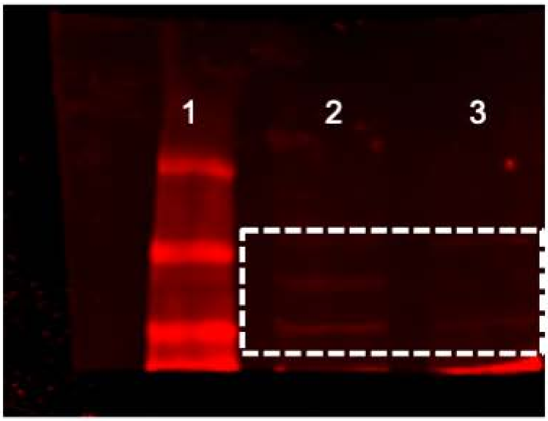

Total Protein original western blot:
Protein ladder lane 1
siNC BHPrS1 lane 2
siZC3H4 BHPrS1 lane 3

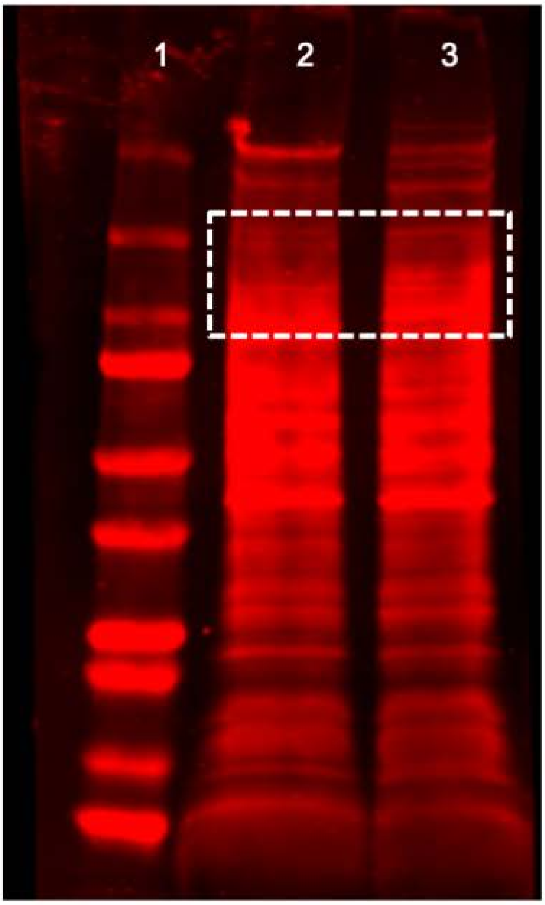

Original data: E-Cadherin Western blot from Figure 6 and Supplemental Figure S4

E-cadherin original western blot:
Protein ladder lane 1
BPH-1 lane 2 (positive control)
siNC BHPrS1 lane 3
siZC3H4 BHPrS1 lane 4

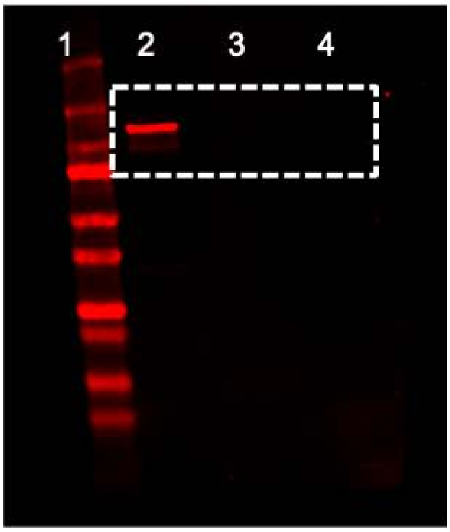

Total Protein for E-cadherin original western blot:
Protein ladder lane 1
BPH-1 lane 2 (positive control)
siNC BHPrS1 lane 3
siZC3H4 BHPrS1 lane 4

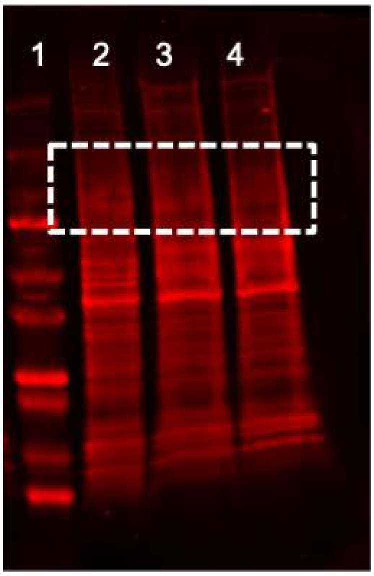

Original data: N-cadherin Western blot from Figure 6 and Supplemental Figure S4

N-cadherin original
western blot:
Protein ladder lane 1
siNC BHPrS1 lane 2
siZC3H4 BHPrS1 lane 3

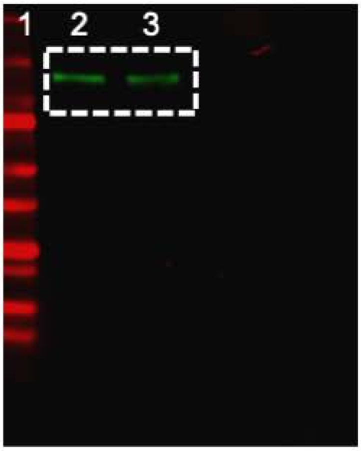

Total Protein for N-cadherin original
western blot:
Protein ladder lane 1
siNC BHPrS1 lane 2
siZC3H4 BHPrS1 lane 3

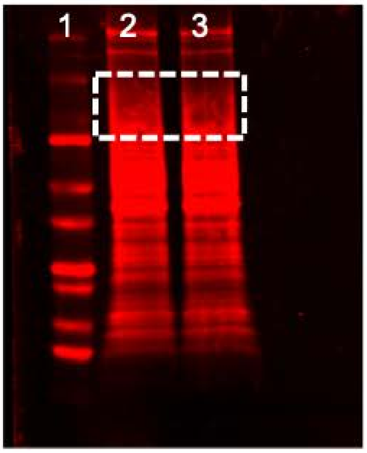

Original data: Vimentin Western blot from Figure 6 and Supplemental Figure S4

Vimentin original western blot:
Protein ladder lane 1
BPH-1 lane 2
siNC BHPrS1 lane 3
siZC3H4 BHPrS1 lane 4

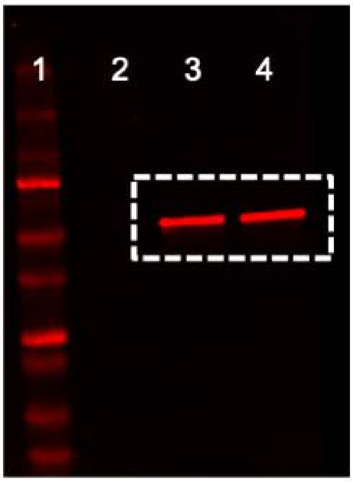

Total Protein for Vimentin original western blot:
Protein ladder lane 1
BPH-1 lane 2
siNC BHPrS1 lane 3
siZC3H4 BHPrS1 lane 4

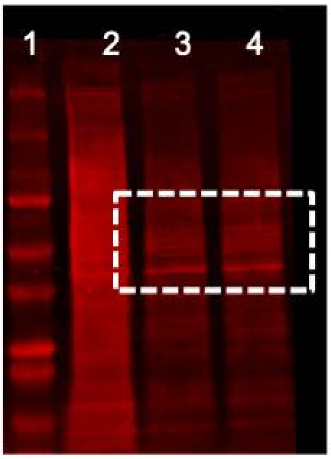

Original data: PCR Quantification Cq Results Files

From Figure 2 and Supplemental Figure S2
Cytokine analysis of BHPrS1 cells treated with glucose or galactose with and without 50 nM rotenone

	A	B	C	D	E	F	H	I	J
1		Well	Fluor	Target	Content	Sample	Cq	Cq Mean	Cq Std. Dev
2		A01	SYBR	YWHAZ	Unkn	GLU1	22.17	22.17	0.000
3		A02	SYBR	YWHAZ	Unkn	GLU1	22.14	22.14	0.000
4		A03	SYBR	IL1b	Unkn	GLU1	30.07	30.07	0.000
5		A04	SYBR	IL1b	Unkn	GLU1	29.20	29.20	0.000
6		A05	SYBR	IL6	Unkn	GLU1	29.71	29.71	0.000
7		A06	SYBR	IL6	Unkn	GLU1	28.12	28.12	0.000
8		A07	SYBR	CXCL1	Unkn	GLU1		0.00	0.000
9		A08	SYBR	CXCL1	Unkn	GLU1	37.49	37.49	0.000
10		A09	SYBR	TNF	Unkn	GLU1	37.71	37.71	0.000
11		A10	SYBR	TNF	Unkn	GLU1	37.18	37.18	0.000
12		A11	SYBR		Unkn			0.00	0.000
13		A12	SYBR		Unkn			0.00	0.000
14		B01	SYBR	YWHAZ	Unkn	GLU2	20.94	20.94	0.000
15		B02	SYBR	YWHAZ	Unkn	GLU2	20.37	20.37	0.000
16		B03	SYBR	IL1b	Unkn	GLU2	28.65	28.65	0.000
17		B04	SYBR	IL1b	Unkn	GLU2	28.98	28.98	0.000
18		B05	SYBR	IL6	Unkn	GLU2	28.76	28.76	0.000
19		B06	SYBR	IL6	Unkn	GLU2	28.26	28.26	0.000
20		B07	SYBR	CXCL1	Unkn	GLU2	32.28	32.28	0.000
21		B08	SYBR	CXCL1	Unkn	GLU2	30.31	30.31	0.000
22		B09	SYBR	TNF	Unkn	GLU2	36.22	36.22	0.000
23		B10	SYBR	TNF	Unkn	GLU2	36.20	36.20	0.000
24		B11	SYBR		Unkn			0.00	0.000
25		B12	SYBR		Unkn			0.00	0.000
26		C01	SYBR	YWHAZ	Unkn	GLU3	20.12	20.12	0.000
27		C02	SYBR	YWHAZ	Unkn	GLU3	19.86	19.86	0.000
28		C03	SYBR	IL1b	Unkn	GLU3	27.82	27.82	0.000
29		C04	SYBR	IL1b	Unkn	GLU3	27.49	27.49	0.000
30		C05	SYBR	IL6	Unkn	GLU3	27.40	27.40	0.000
31		C06	SYBR	IL6	Unkn	GLU3	27.15	27.15	0.000
32		C07	SYBR	CXCL1	Unkn	GLU3	32.67	32.67	0.000
33		C08	SYBR	CXCL1	Unkn	GLU3	34.14	34.14	0.000
34		C09	SYBR	TNF	Unkn	GLU3	36.07	36.07	0.000
35		C10	SYBR	TNF	Unkn	GLU3	35.54	35.54	0.000
36		C11	SYBR		Unkn			0.00	0.000
37		C12	SYBR		Unkn			0.00	0.000
38		D01	SYBR	YWHAZ	Unkn	GLU ROT1	18.00	18.00	0.000
39		D02	SYBR	YWHAZ	Unkn	GLU ROT1	17.84	17.84	0.000
40		D03	SYBR	IL1b	Unkn	GLU ROT1	25.79	25.79	0.000
41		D04	SYBR	IL1b	Unkn	GLU ROT1	26.04	26.04	0.000
42		D05	SYBR	IL6	Unkn	GLU ROT1	24.86	24.86	0.000
43		D06	SYBR	IL6	Unkn	GLU ROT1	24.57	24.57	0.000
44		D07	SYBR	CXCL1	Unkn	GLU ROT1	29.77	29.77	0.000
45		D08	SYBR	CXCL1	Unkn	GLU ROT1	29.25	29.25	0.000
46		D09	SYBR	TNF	Unkn	GLU ROT1	34.95	34.95	0.000
47		D10	SYBR	TNF	Unkn	GLU ROT1	35.32	35.32	0.000
48		D11	SYBR		Unkn			0.00	0.000
49		D12	SYBR		Unkn			0.00	0.000
50		E01	SYBR	YWHAZ	Unkn	GLU ROT2	18.63	18.63	0.000
51		E02	SYBR	YWHAZ	Unkn	GLU ROT2	18.29	18.29	0.000

	A	B	C	D	E	F	H	I	J
52		E03	SYBR	IL1b	Unkn	GLU ROT2	27.57	27.57	0.000
53		E04	SYBR	IL1b	Unkn	GLU ROT2	27.06	27.06	0.000
54		E05	SYBR	IL6	Unkn	GLU ROT2	25.42	25.42	0.000
55		E06	SYBR	IL6	Unkn	GLU ROT2	25.42	25.42	0.000
56		E07	SYBR	CXCL1	Unkn	GLU ROT2	33.06	33.06	0.000
57		E08	SYBR	CXCL1	Unkn	GLU ROT2	33.97	33.97	0.000
58		E09	SYBR	TNF	Unkn	GLU ROT2	37.22	37.22	0.000
59		E10	SYBR	TNF	Unkn	GLU ROT2	36.44	36.44	0.000
60		E11	SYBR		Unkn			0.00	0.000
61		E12	SYBR		Unkn			0.00	0.000
62		F01	SYBR	YWHAZ	Unkn	GLU ROT3	19.07	19.07	0.000
63		F02	SYBR	YWHAZ	Unkn	GLU ROT3	19.04	19.04	0.000
64		F03	SYBR	IL1b	Unkn	GLU ROT3	27.68	27.68	0.000
65		F04	SYBR	IL1b	Unkn	GLU ROT3	27.21	27.21	0.000
66		F05	SYBR	IL6	Unkn	GLU ROT3	26.61	26.61	0.000
67		F06	SYBR	IL6	Unkn	GLU ROT3	26.44	26.44	0.000
68		F07	SYBR	CXCL1	Unkn	GLU ROT3	36.98	36.98	0.000
69		F08	SYBR	CXCL1	Unkn	GLU ROT3	35.50	35.50	0.000
70		F09	SYBR	TNF	Unkn	GLU ROT3	37.97	37.97	0.000
71		F10	SYBR	TNF	Unkn	GLU ROT3	38.22	38.22	0.000
72		F11	SYBR		Unkn			0.00	0.000
73		F12	SYBR		Unkn			0.00	0.000
74		G01	SYBR		Unkn			0.00	0.000
75		G02	SYBR		Unkn			0.00	0.000
76		G03	SYBR		Unkn			0.00	0.000
77		G04	SYBR		Unkn			0.00	0.000
78		G05	SYBR		Unkn			0.00	0.000
79		G06	SYBR		Unkn			0.00	0.000
80		G07	SYBR		Unkn			0.00	0.000
81		G08	SYBR		Unkn			0.00	0.000
82		G09	SYBR		Unkn			0.00	0.000
83		G10	SYBR		Unkn			0.00	0.000
84		G11	SYBR		Unkn			0.00	0.000
85		G12	SYBR		Unkn			0.00	0.000
86		H01	SYBR		Unkn			0.00	0.000
87		H02	SYBR		Unkn			0.00	0.000
88		H03	SYBR		Unkn			0.00	0.000
89		H04	SYBR		Unkn			0.00	0.000
90		H05	SYBR		Unkn			0.00	0.000
91		H06	SYBR		Unkn			0.00	0.000
92		H07	SYBR		Unkn			0.00	0.000
93		H08	SYBR		Unkn			0.00	0.000
94		H09	SYBR		Unkn			0.00	0.000
95		H10	SYBR		Unkn			0.00	0.000
96		H11	SYBR		Unkn			0.00	0.000
97		H12	SYBR		Unkn			0.00	0.000

	A	B	C	D	E	F	H	I	J
1		Well	Fluor	Target	Content	Sample	Cq	Cq Mean	Cq Std. Dev
2		A01	SYBR	YWHAZ	Unkn	GAL1	18.32	18.32	0.000
3		A02	SYBR	YWHAZ	Unkn	GAL1	18.19	18.19	0.000
4		A03	SYBR	IL1b	Unkn	GAL1	25.68	25.68	0.000
5		A04	SYBR	IL1b	Unkn	GAL1	25.26	25.26	0.000
6		A05	SYBR	IL6	Unkn	GAL1	22.66	22.66	0.000
7		A06	SYBR	IL6	Unkn	GAL1	22.51	22.51	0.000
8		A07	SYBR	CXCL1	Unkn	GAL1	24.75	24.75	0.000
9		A08	SYBR	CXCL1	Unkn	GAL1	24.72	24.72	0.000
10		A09	SYBR	TNF	Unkn	GAL1	34.42	34.42	0.000
11		A10	SYBR	TNF	Unkn	GAL1	35.40	35.40	0.000
12		A11	SYBR		Unkn			0.00	0.000
13		A12	SYBR		Unkn			0.00	0.000
14		B01	SYBR	YWHAZ	Unkn	GAL2	18.29	18.29	0.000
15		B02	SYBR	YWHAZ	Unkn	GAL2	18.05	18.05	0.000
16		B03	SYBR	IL1b	Unkn	GAL2	26.10	26.10	0.000
17		B04	SYBR	IL1b	Unkn	GAL2	26.01	26.01	0.000
18		B05	SYBR	IL6	Unkn	GAL2	24.15	24.15	0.000
19		B06	SYBR	IL6	Unkn	GAL2	23.71	23.71	0.000
20		B07	SYBR	CXCL1	Unkn	GAL2	26.66	26.66	0.000
21		B08	SYBR	CXCL1	Unkn	GAL2	26.46	26.46	0.000
22		B09	SYBR	TNF	Unkn	GAL2	35.24	35.24	0.000
23		B10	SYBR	TNF	Unkn	GAL2	35.41	35.41	0.000
24		B11	SYBR		Unkn			0.00	0.000
25		B12	SYBR		Unkn			0.00	0.000
26		C01	SYBR	YWHAZ	Unkn	GAL3	19.29	19.29	0.000
27		C02	SYBR	YWHAZ	Unkn	GAL3	19.05	19.05	0.000
28		C03	SYBR	IL1b	Unkn	GAL3	26.44	26.44	0.000
29		C04	SYBR	IL1b	Unkn	GAL3	26.57	26.57	0.000
30		C05	SYBR	IL6	Unkn	GAL3	24.56	24.56	0.000
31		C06	SYBR	IL6	Unkn	GAL3	24.31	24.31	0.000
32		C07	SYBR	CXCL1	Unkn	GAL3	28.92	28.92	0.000
33		C08	SYBR	CXCL1	Unkn	GAL3	29.17	29.17	0.000
34		C09	SYBR	TNF	Unkn	GAL3	35.99	35.99	0.000
35		C10	SYBR	TNF	Unkn	GAL3	36.29	36.29	0.000
36		C11	SYBR		Unkn			0.00	0.000
37		C12	SYBR		Unkn			0.00	0.000
38		D01	SYBR	YWHAZ	Unkn	GAL ROT 1	17.85	17.85	0.000
39		D02	SYBR	YWHAZ	Unkn	GAL ROT 1	18.01	18.01	0.000
40		D03	SYBR	IL1b	Unkn	GAL ROT 1	25.39	25.39	0.000
41		D04	SYBR	IL1b	Unkn	GAL ROT 1	25.31	25.31	0.000
42		D05	SYBR	IL6	Unkn	GAL ROT 1	23.52	23.52	0.000
43		D06	SYBR	IL6	Unkn	GAL ROT 1	23.26	23.26	0.000
44		D07	SYBR	CXCL1	Unkn	GAL ROT 1	27.28	27.28	0.000
45		D08	SYBR	CXCL1	Unkn	GAL ROT 1	26.97	26.97	0.000
46		D09	SYBR	TNF	Unkn	GAL ROT 1	32.16	32.16	0.000
47		D10	SYBR	TNF	Unkn	GAL ROT 1	34.87	34.87	0.000
48		D11	SYBR		Unkn			0.00	0.000
49		D12	SYBR		Unkn			0.00	0.000
50		E01	SYBR	YWHAZ	Unkn	GAL ROT 2	19.39	19.39	0.000
51		E02	SYBR	YWHAZ	Unkn	GAL ROT 2	19.28	19.28	0.000

	A	B	C	D	E	F	H	I	J
52		E03	SYBR	IL1b	Unkn	GAL ROT 2	27.12	27.12	0.000
53		E04	SYBR	IL1b	Unkn	GAL ROT 2	27.07	27.07	0.000
54		E05	SYBR	IL6	Unkn	GAL ROT 2	24.97	24.97	0.000
55		E06	SYBR	IL6	Unkn	GAL ROT 2	24.96	24.96	0.000
56		E07	SYBR	CXCL1	Unkn	GAL ROT 2	31.90	31.90	0.000
57		E08	SYBR	CXCL1	Unkn	GAL ROT 2	32.30	32.30	0.000
58		E09	SYBR	TNF	Unkn	GAL ROT 2	35.14	35.14	0.000
59		E10	SYBR	TNF	Unkn	GAL ROT 2	36.36	36.36	0.000
60		E11	SYBR		Unkn			0.00	0.000
61		E12	SYBR		Unkn			0.00	0.000
62		F01	SYBR	YWHAZ	Unkn	GAL ROT 3	19.59	19.59	0.000
63		F02	SYBR	YWHAZ	Unkn	GAL ROT 3	19.32	19.32	0.000
64		F03	SYBR	IL1b	Unkn	GAL ROT 3	27.09	27.09	0.000
65		F04	SYBR	IL1b	Unkn	GAL ROT 3	26.77	26.77	0.000
66		F05	SYBR	IL6	Unkn	GAL ROT 3	24.29	24.29	0.000
67		F06	SYBR	IL6	Unkn	GAL ROT 3	24.42	24.42	0.000
68		F07	SYBR	CXCL1	Unkn	GAL ROT 3	30.21	30.21	0.000
69		F08	SYBR	CXCL1	Unkn	GAL ROT 3	30.50	30.50	0.000
70		F09	SYBR	TNF	Unkn	GAL ROT 3	34.58	34.58	0.000
71		F10	SYBR	TNF	Unkn	GAL ROT 3	35.21	35.21	0.000
72		F11	SYBR		Unkn			0.00	0.000
73		F12	SYBR		Unkn			0.00	0.000
74		G01	SYBR		Unkn			0.00	0.000
75		G02	SYBR		Unkn			0.00	0.000
76		G03	SYBR		Unkn			0.00	0.000
77		G04	SYBR		Unkn			0.00	0.000
78		G05	SYBR		Unkn			0.00	0.000
79		G06	SYBR		Unkn			0.00	0.000
80		G07	SYBR		Unkn			0.00	0.000
81		G08	SYBR		Unkn			0.00	0.000
82		G09	SYBR		Unkn			0.00	0.000
83		G10	SYBR		Unkn			0.00	0.000
84		G11	SYBR		Unkn			0.00	0.000
85		G12	SYBR		Unkn			0.00	0.000
86		H01	SYBR		Unkn			0.00	0.000
87		H02	SYBR		Unkn			0.00	0.000
88		H03	SYBR		Unkn			0.00	0.000
89		H04	SYBR		Unkn			0.00	0.000
90		H05	SYBR		Unkn			0.00	0.000
91		H06	SYBR		Unkn			0.00	0.000
92		H07	SYBR		Unkn			0.00	0.000
93		H08	SYBR		Unkn			0.00	0.000
94		H09	SYBR		Unkn			0.00	0.000
95		H10	SYBR		Unkn			0.00	0.000
96		H11	SYBR		Unkn			0.00	0.000
97		H12	SYBR		Unkn			0.00	0.000

Original data: PCR Quantification Cq Results Files

From Figure 4
ZC3H4 analysis of BHPrS1 cells treated with siNC and siZC3H4.1 and siZC3H4.3

	A	B	C	D	E	F	H	I	J
1		Well	Fluor	Target	Content	Sample	Cq	Cq Mean	Cq Std. Dev
2		A01	SYBR	YWHAZ	Unkn	NC1	22.20	22.20	0.000
3		A02	SYBR	YWHAZ	Unkn	NC2	21.86	21.86	0.000
4		A03	SYBR	YWHAZ	Unkn	NC3	21.77	21.77	0.000
5		A04	SYBR	YWHAZ	Unkn	Z1-1	21.94	21.94	0.000
6		A05	SYBR	YWHAZ	Unkn	Z1-2	21.99	21.99	0.000
7		A06	SYBR	YWHAZ	Unkn	Z1-3	22.18	22.18	0.000
8		A07	SYBR	YWHAZ	Unkn	Z2-1	21.68	21.68	0.000
9		A08	SYBR	YWHAZ	Unkn	Z2-2	21.64	21.64	0.000
10		A09	SYBR	YWHAZ	Unkn	Z2-3		0.00	0.000
11		A10	SYBR	YWHAZ	Unkn	Z3-1	21.31	21.31	0.000
12		A11	SYBR	YWHAZ	Unkn	Z3-2	21.85	21.85	0.000
13		A12	SYBR	YWHAZ	Unkn	Z3-3	26.34	26.34	0.000
14		B01	SYBR	YWHAZ	Unkn	NC1	22.28	22.28	0.000
15		B02	SYBR	YWHAZ	Unkn	NC2	21.77	21.77	0.000
16		B03	SYBR	YWHAZ	Unkn	NC3	21.57	21.57	0.000
17		B04	SYBR	YWHAZ	Unkn	Z1-1	21.97	21.97	0.000
18		B05	SYBR	YWHAZ	Unkn	Z1-2	22.05	22.05	0.000
19		B06	SYBR	YWHAZ	Unkn	Z1-3	22.03	22.03	0.000
20		B07	SYBR	YWHAZ	Unkn	Z2-1	21.63	21.63	0.000
21		B08	SYBR	YWHAZ	Unkn	Z2-2	21.45	21.45	0.000
22		B09	SYBR	YWHAZ	Unkn	Z2-3		0.00	0.000
23		B10	SYBR	YWHAZ	Unkn	Z3-1	21.37	21.37	0.000
24		B11	SYBR	YWHAZ	Unkn	Z3-2	21.77	21.77	0.000
25		B12	SYBR	YWHAZ	Unkn	Z3-3	26.37	26.37	0.000
26		C01	SYBR	ZC3H4	Unkn	NC1	29.22	29.22	0.000
27		C02	SYBR	ZC3H5	Unkn	NC2	29.29	29.29	0.000
28		C03	SYBR	ZC3H6	Unkn	NC3	29.06	29.06	0.000
29		C04	SYBR	ZC3H7	Unkn	Z1-1	30.30	30.30	0.000
30		C05	SYBR	ZC3H8	Unkn	Z1-2	30.88	30.88	0.000
31		C06	SYBR	ZC3H9	Unkn	Z1-3	31.54	31.54	0.000
32		C07	SYBR	ZC3H10	Unkn	Z2-1	30.61	30.61	0.000
33		C08	SYBR	ZC3H11	Unkn	Z2-2	31.65	31.65	0.000
34		C09	SYBR	ZC3H12	Unkn	Z2-3		0.00	0.000
35		C10	SYBR	ZC3H13	Unkn	Z3-1	32.23	32.23	0.000
36		C11	SYBR	ZC3H14	Unkn	Z3-2	33.04	33.04	0.000
37		C12	SYBR	ZC3H15	Unkn	Z3-3	35.93	35.93	0.000
38		D01	SYBR	ZC3H16	Unkn	NC1	29.38	29.38	0.000
39		D02	SYBR	ZC3H17	Unkn	NC2	29.51	29.51	0.000
40		D03	SYBR	ZC3H18	Unkn	NC3	29.14	29.14	0.000
41		D04	SYBR	ZC3H19	Unkn	Z1-1	30.42	30.42	0.000
42		D05	SYBR	ZC3H20	Unkn	Z1-2	30.84	30.84	0.000
43		D06	SYBR	ZC3H21	Unkn	Z1-3	31.60	31.60	0.000
44		D07	SYBR	ZC3H22	Unkn	Z2-1	30.54	30.54	0.000
45		D08	SYBR	ZC3H23	Unkn	Z2-2	31.80	31.80	0.000
46		D09	SYBR	ZC3H24	Unkn	Z2-3		0.00	0.000
47		D10	SYBR	ZC3H25	Unkn	Z3-1	32.07	32.07	0.000
48		D11	SYBR	ZC3H26	Unkn	Z3-2	33.20	33.20	0.000
49		D12	SYBR	ZC3H27	Unkn	Z3-3	37.65	37.65	0.000
50		E01	SYBR	N-CAD	Unkn	NC1	24.62	24.62	0.000
51		E02	SYBR	N-CAD	Unkn	NC2	24.36	24.36	0.000

	A	B	C	D	E	F	H	I	J
52		E03	SYBR	N-CAD	Unkn	NC3	24.56	24.56	0.000
53		E04	SYBR	N-CAD	Unkn	Z1-1	24.45	24.45	0.000
54		E05	SYBR	N-CAD	Unkn	Z1-2	24.68	24.68	0.000
55		E06	SYBR	N-CAD	Unkn	Z1-3	24.90	24.90	0.000
56		E07	SYBR	N-CAD	Unkn	Z2-1	24.39	24.39	0.000
57		E08	SYBR	N-CAD	Unkn	Z2-2	24.32	24.32	0.000
58		E09	SYBR	N-CAD	Unkn	Z2-3		0.00	0.000
59		E10	SYBR	N-CAD	Unkn	Z3-1	24.00	24.00	0.000
60		E11	SYBR	N-CAD	Unkn	Z3-2	24.43	24.43	0.000
61		E12	SYBR	N-CAD	Unkn	Z3-3	27.95	27.95	0.000
62		F01	SYBR	N-CAD	Unkn	NC1	24.87	24.87	0.000
63		F02	SYBR	N-CAD	Unkn	NC2	24.73	24.73	0.000
64		F03	SYBR	N-CAD	Unkn	NC3	24.79	24.79	0.000
65		F04	SYBR	N-CAD	Unkn	Z1-1	24.68	24.68	0.000
66		F05	SYBR	N-CAD	Unkn	Z1-2	24.82	24.82	0.000
67		F06	SYBR	N-CAD	Unkn	Z1-3	25.08	25.08	0.000
68		F07	SYBR	N-CAD	Unkn	Z2-1	24.49	24.49	0.000
69		F08	SYBR	N-CAD	Unkn	Z2-2	24.32	24.32	0.000
70		F09	SYBR	N-CAD	Unkn	Z2-3		0.00	0.000
71		F10	SYBR	N-CAD	Unkn	Z3-1	24.02	24.02	0.000
72		F11	SYBR	N-CAD	Unkn	Z3-2	24.62	24.62	0.000
73		F12	SYBR	N-CAD	Unkn	Z3-3	27.60	27.60	0.000
74		G01	SYBR		Unkn			0.00	0.000
75		G02	SYBR		Unkn			0.00	0.000
76		G03	SYBR		Unkn			0.00	0.000
77		G04	SYBR		Unkn			0.00	0.000
78		G05	SYBR		Unkn			0.00	0.000
79		G06	SYBR		Unkn			0.00	0.000
80		G07	SYBR		Unkn			0.00	0.000
81		G08	SYBR		Unkn			0.00	0.000
82		G09	SYBR		Unkn			0.00	0.000
83		G10	SYBR		Unkn			0.00	0.000
84		G11	SYBR		Unkn			0.00	0.000
85		G12	SYBR		Unkn			0.00	0.000
86		H01	SYBR		Unkn			0.00	0.000
87		H02	SYBR		Unkn			0.00	0.000
88		H03	SYBR		Unkn			0.00	0.000
89		H04	SYBR		Unkn			0.00	0.000
90		H05	SYBR		Unkn			0.00	0.000
91		H06	SYBR		Unkn			0.00	0.000
92		H07	SYBR		Unkn			0.00	0.000
93		H08	SYBR		Unkn			0.00	0.000
94		H09	SYBR		Unkn			0.00	0.000
95		H10	SYBR		Unkn			0.00	0.000
96		H11	SYBR		Unkn			0.00	0.000
97		H12	SYBR		Unkn			0.00	0.000
